# Supplementary material for: Modular DNA strand-displacement controllers for directing material expansion
Source: Nat Commun. 2018 Sep 14;9:3766. doi: 10.1038/s41467-018-06218-w (PMC6138645; doi:10.1038/s41467-018-06218-w)
Supplement: Supplementary file 1 — Supplementary Information [file 41467_2018_6218_MOESM1_ESM.pdf]

Supplementary Information for

Modular DNA Strand-Displacement Controllers for Directing Material Expansion

Joshua Fern[1] *and Rebecca Schulman*[1,2]

[1]Chemical and Biomolecular Engineering, Johns Hopkins University, Baltimore, MD 21218; United States of America; [2]Computer Science, Johns Hopkins University, Baltimore, MD 21218, United States of America

Supplementary Methods:

DNA sequence design

The nucleic acid thermodynamic modeling and design software NUPACK[1] was used to design sequences for the locking and unlocking mechanism and for parts of sequences of the logic circuit and ATP sensor that were not taken from previous literature (Supplementary Figs. 1, 2, 18, and 22 and Supplementary Table 1).[2–5] During each design process, NUPACK's design process was run multiple times to give multiple potential sequence sets. The optimum sequence set for the locked crosslink from the pool of sequences NUPACK produced was chosen such that at equilibrium NUPACK predicted minimal to no interaction between the hairpins H_1/H_2 and the locked crosslink with the hairpins at 10x the concentration of the locked crosslink strands (A, R, and the lock set to 1 µM each). The toehold lengths for the Catalyst/Key binding to the locked crosslink and the Helper binding Catalyst-locked crosslink intermediate complex ("s" and "t" domains in Supplementary Figs. 1 and 2) were chosen to match toehold lengths in previously demonstrated catalytic circuits.[5–7] Given the well-characterized energetics of toehold-mediated strand-displacement,[8,9] the length of the toehold for Helper strand binding needed to be longer than the toehold for the Catalyst strand in order for the Catalyst to be efficiently released from the intermediate complex by the Helper strand.

The additional sequences designed for the ATP sensing and logic-based controllers were designed to be sequence restricted (*e.g.*, to contain only 3 nucleotides – A, G, and C) and designed against the sequences of the other components (*e.g.*, locking mechanism, hairpins, Catalyst/Helper) such that there was minimal nonspecific hybridization between components.

PAGE protocol

Polyacrylamide gel electrophoresis (PAGE) was used to characterize the rate of spurious interactions between the strands constituting gel crosslinks, locks, helper strands, and other controller components. Polyacrylamide gels were cast at 10% acrylamide using 19:1 40% acrylamide/bis solution (Bio-Rad) with ammonium persulfate (APS) and tetramethylenediamine (TEMED) as the polymerization initiator and catalyst. All gels were run for 1.5 hours at 150 volts with TAEM as the running buffer. After running, the gels were stained for 30 minutes with 1x SYBR Gold (Invitrogen) for DNA visualization. Gels were imaged using a Syngene GBox EF2 gel imager.

Stocks of DNA complexes (AR, ARL, ARtTL, ARLnl, and HelperNT-LnL) were each annealed at 1 µM in TAEM buffer from 90 to 20 °C using an Eppendorf PCR at 1 °C/minute. The hairpin H_1 was flash cooled on ice after heating to 95 °C at a stock concentration of 10 µM. Reaction mixtures (*e.g.*, AR + H_1) were incubated at 300 nM per species overnight at room temperature immediately prior to the PAGE run. Loading dye was added at a ratio of 5:1 to the reaction mixture and 10 µL of the total solution was added to the specified wells.

Supplementary Note 1: Design of lockable DNA crosslinks

To design DNA crosslinks that could be in either an active state where DNA hairpins incorporated into the crosslink can trigger hydrogel expansion, or an inactive state, where hairpins are unable to interact with the crosslinks, we began with one of the sequences for hydrogel crosslinks used in Cangialosi *et al* (Supplementary Figs. 1 and 2, Supplementary Table 1).[2] To create a locking mechanism for this crosslink, we modified the sequences of the crosslink to allow a locking strand to hybridize to the crosslink duplex by extending the sequences of

crosslinks on the 5' terminus of the *A* strand and the 3' terminus of the *R* strand (Supplementary Methods). We termed this the unlocked complex (Supplementary Fig. 1).

The locking strand that we first designed for this crosslink, *GblHPCCnl*, was simply the exact complement of the toeholds for the hairpin and the extended sequences of the crosslinks. However, we found using a PAGE gel (Supplementary Methods) that when the unlocked complex was locked with *GblHPCCnl* to produce the locked complex *ARLnl*, the complex still interacted significantly with Helper strand *HelperNTlHPCC* (Supplementary Fig. 3). As a result, we would expect significant swelling of a gel using these crosslinks even when no Catalyst signal is present. The ensemble equilibrium structures of the strands of this locked complex, as predicted by the nucleic acid thermodynamic modeling software NUPACK,[1] showed that the bases where the *A*, *R*, and locking strand *GblHPCCnl* joined within the locked complex *ARLnl* only had a 50% probability of being hybridized at room temperature (Supplementary Fig. 4). We hypothesized that adding unpaired nucleotides to the locking strand at this junction would reduce the strain on the paired bases at that location and increase the potential for hybridization of these bases. This improved hybridization would then reduce the potential for initiation of helper-complex hybridization at the junction. Analysis using NUPACK supported this hypothesis, suggesting an increase in equilibrium hybridization probability of the bases at the junction after the addition of a single base to the locking strand, named *GblHPCC* (Supplementary Fig. 4).

Equilibrium analysis with NUPACK also suggested that the amount of Helper strands *HelperNTlHPCC* hybridized to the locking strand in the presence of the crosslink strands is potentially too large, which would ultimately lead to undesired unlocking of the locked complexes in the absence of Catalyst strands. Thus, we decreased the number of bases in the

Helper strand that was complementary to the locking strand by one base on the 3' terminus (Supplementary Fig. 1, Supplementary Table 1).

We used PAGE to experimentally characterize the potential for the truncated Helper strand *HelperlHPCC* to interact with the locked complex *ARL* and the degree to which unlocking occurred in the presence of both Helper and Catalyst. We found that the inclusion of a nucleotide loop in the junction of the ARL complex (L domain in Supplementary Figs. 1 and 2) and the truncation of the Helper strand together decreased the propensity for interactions between the Helper and ARL complex in the absence of Catalyst (Supplementary Fig. 5). We also found that increasing the number of nucleotides in the loop of the locking strand beyond 1 nucleotide decreased the amount of ARL complex that could be unlocked as shown by an increase in the intensity of the ARL band, and a decrease in the intensity of the AR-H_1 band in the presence of Helper, Catalyst, and H_1.

Because the crosslinks were used in millimolar concentrations, they could not be both purified after synthesis and obtained at large scale. As a result, we expect that a significant number of crosslinks would have synthesis errors. We hypothesized that adding 3 thymine nucleotides to the 5' terminus of the A strand and 3' terminus of the R strand (Supplementary Figs. 1-2) would reduce the amount of interaction between the Helper strand and the locking strand in the ARL complex by decreasing the probability that an error during synthesis at the termini of the A and R strands would affect the base pairing with the locking strand at the duplex termini where the Helper strand could potentially interact. The addition of these bases also slightly increases the hybridization energy at the termini by the addition of single-stranded overhangs as predicted by the nearest-neighbor model for DNA hybridization.[10] A PAGE gel of this thymine-modified complex (*ARtTL*) showed a slight decrease in the degree of spurious

interaction between the Helper and locked complex relative to the degree of interaction between the Helper and the unmodified crosslink strands after crosslinks were incubated in the presence of both Helper and Catalyst in an overnight reaction (Supplementary Fig. 6). The thymine-modified A and R strands, the locking strand with a 1 nucleotide loop, and the 1bp truncated Helper strand were chosen as the optimal set of strands for incorporation into hydrogels (Supplementary Fig. 2).

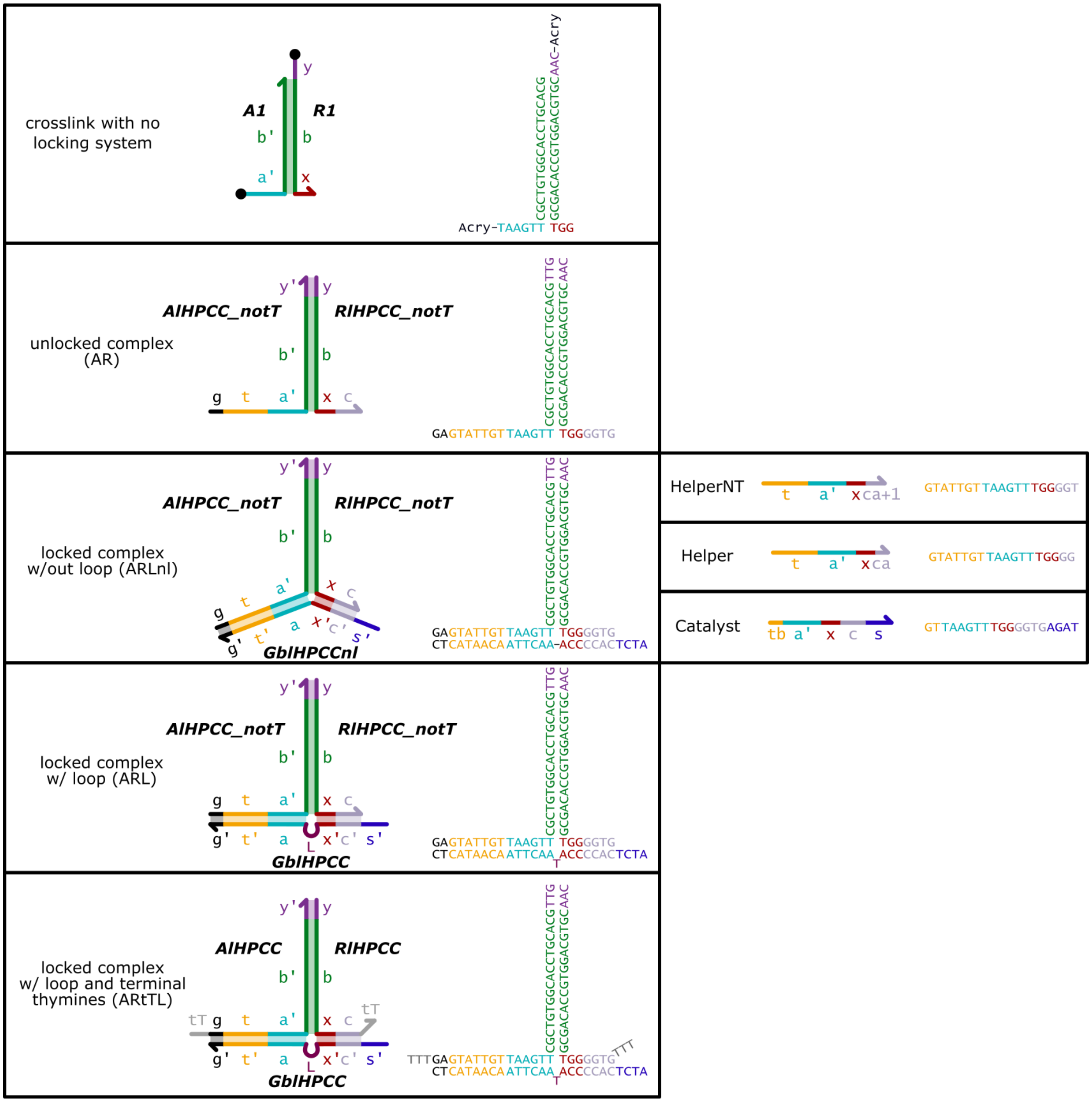

Supplementary Figure 1: DNA strands and complexes considered during the design of the crosslink lock, Helper, and Catalyst. The crosslink without a locking system was used in Cangialosi *et al*.[2] The names listed in parentheses are used in Supplementary Figs. 3 – 6. The hairpin monomer H_1 inserts into the AR complexes by initially binding to the toehold domains *a'* and *x* (teal and red domains). Sequences of each strand in this table are also listed in Supplementary Table 1.

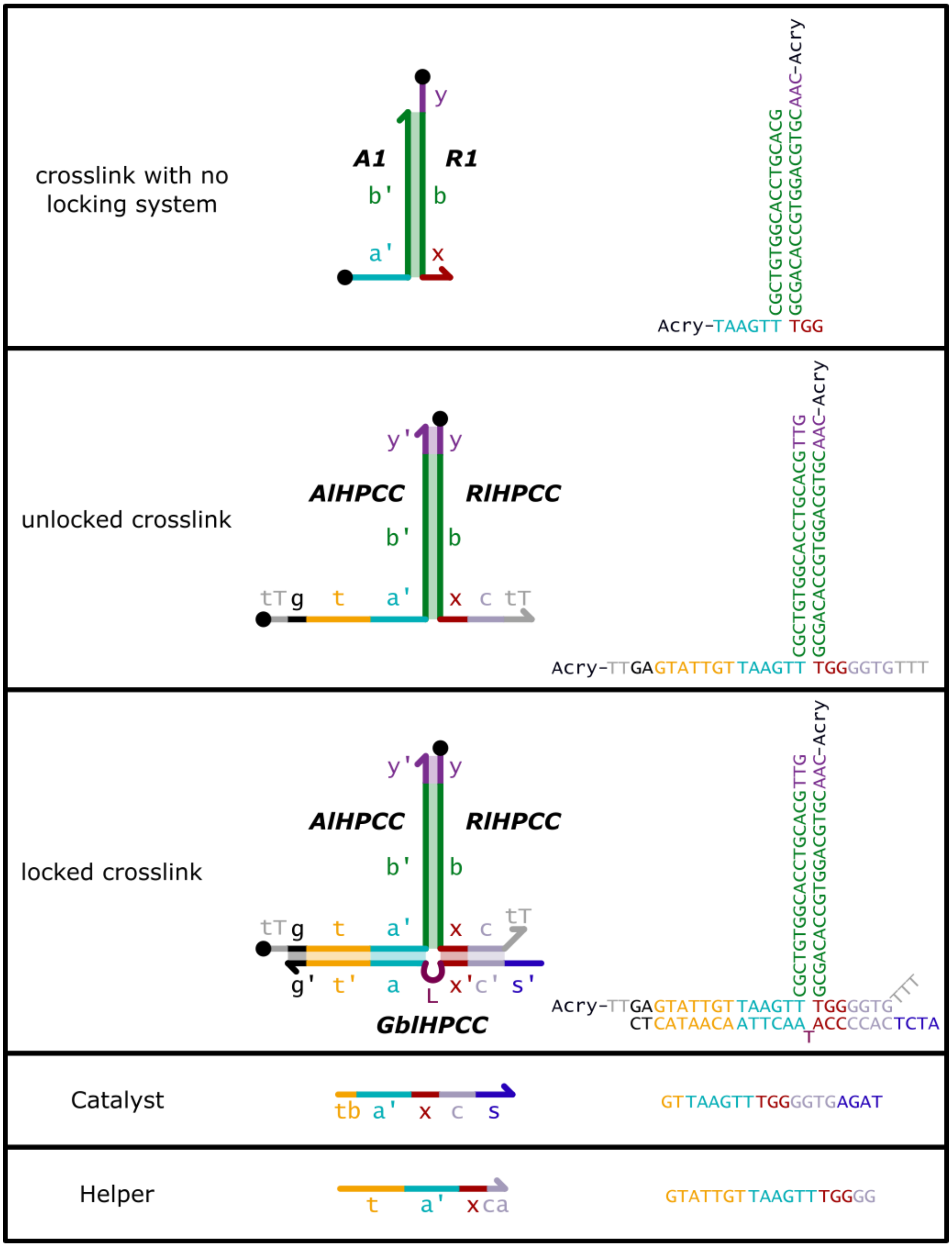

Supplementary Figure 2: Optimized DNA crosslink, Catalyst, and Helper sequences. The crosslink without a locking system is also used in Cangialosi *et al.*[2] The hairpin monomers H_1 or H_{1Term} insert into the crosslink through initial interaction with the domains *a'* and *x* (teal and red domains). Acrydite modifications at the 5' termini of crosslinks are denoted by black circles. Sequences are also given in Supplementary Table 1.

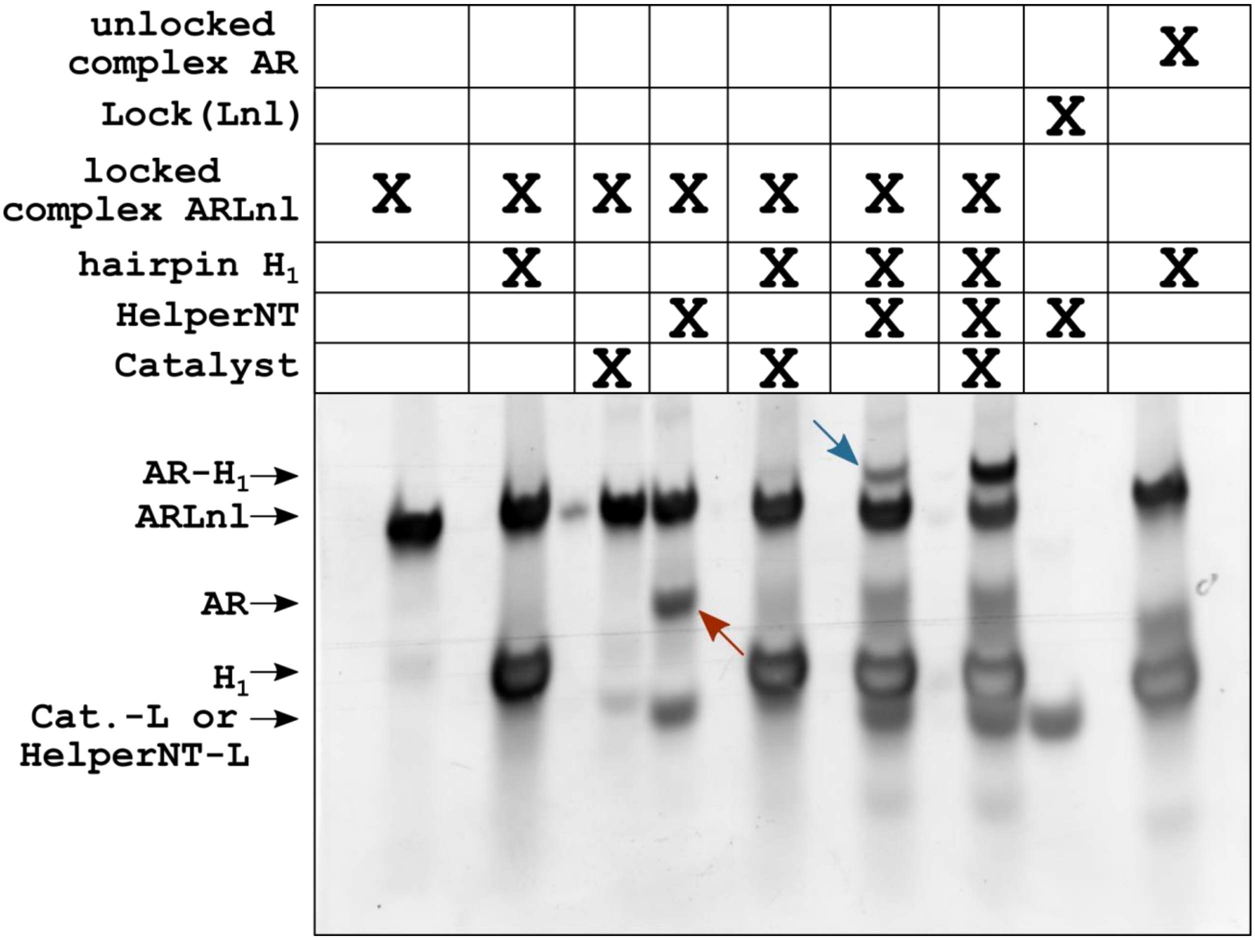

Supplementary Figure 3: PAGE gel showing substantial undesired interactions between the untruncated Helper (HelperNT) and the locking strands without a loop (Lnl). An "X" above each lane denotes the presence of that species in the reaction solution added to that lane. Experiment conducted as described in Supplementary Methods. The brown arrow points to the band containing AR complex unlocked because of undesired interactions between HelperNT and ARLnl complex. The blue arrow points to the AR-H1 complex produced when the crosslinks unlocked as a result of the same HelperNT/ARLnl interaction react with a hairpin. The sequence names for each species, as listed in Supplementary Table 1, are: unlocked complex AR (AlHPCC_notT_noAc, RlHPCC_notT_noAc); Lock (GblHPCCnl); locked complex ARLnl (AlHPCC_notT_noAc, RlHPCC_notT_noAc, GblHPCCnl); hairpin H_1 (H1); HelperNT (HelperNTlHPCC); Catalyst (CatalystlHPCC).

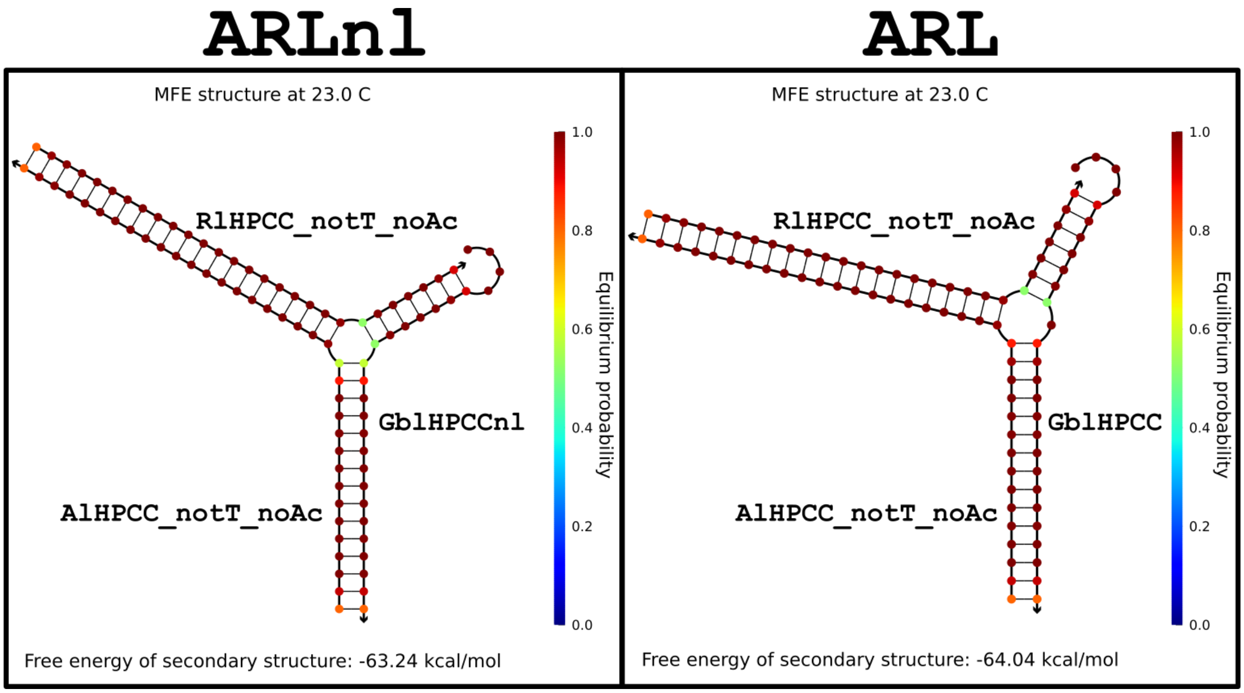

Supplementary Figure 4: Equilibrium structures of the DNA crosslinks in free solution (*i.e.*, not integrated into a hydrogel) predicted by NUPACK.[1] The simulation parameters were 51 mM Na^+ and 12.5 mM Mg^{2+} ions, temperature at 23 °C, and the "some dangles" setting. Sequences are shown in Supplementary Fig. 1 and listed in Supplementary Table 1. Strand names are listed next to each strand.

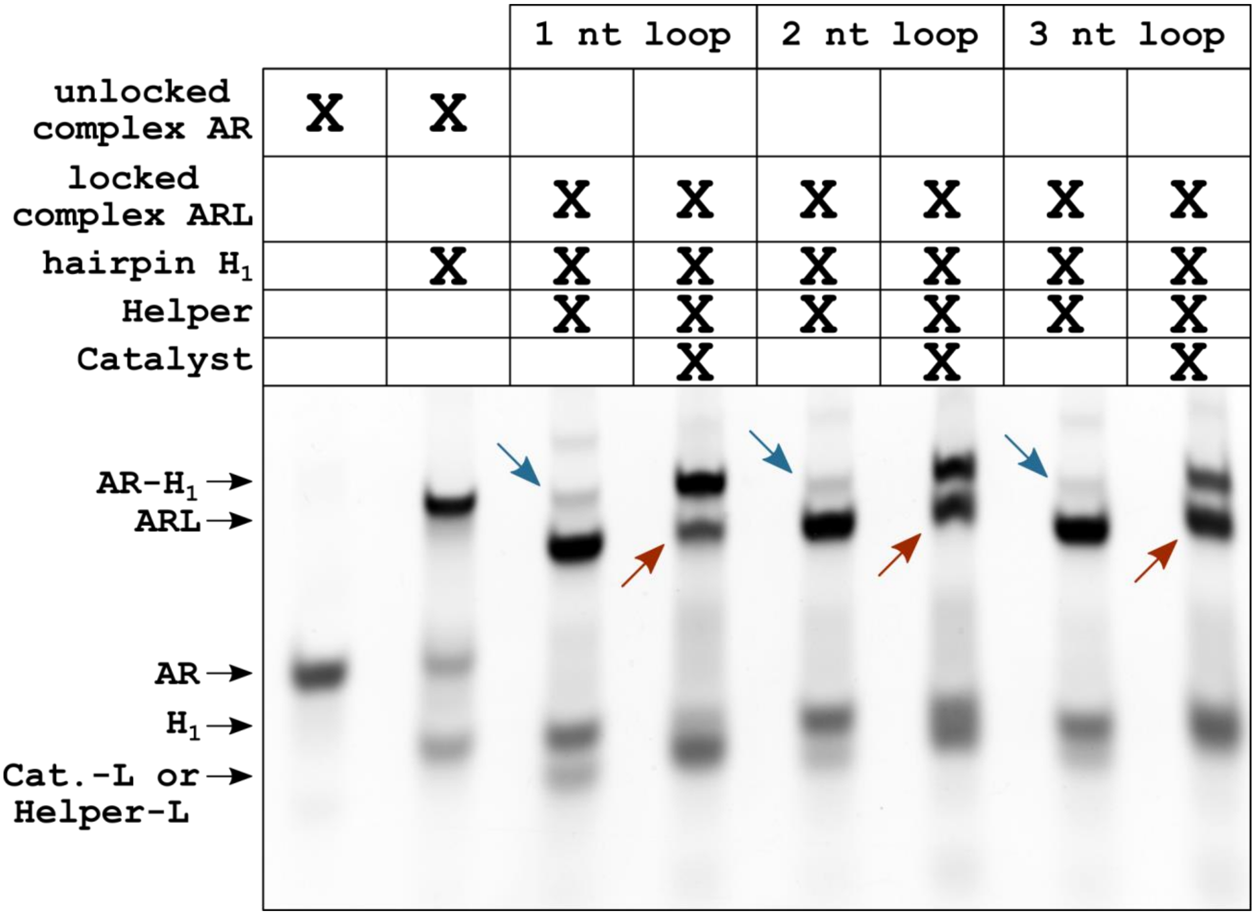

Supplementary Figure 5: PAGE gel demonstrating the effect of adding unpaired nucleotides to the locking strand at the A-R-L junction on the types of interactions between crosslinks, locks, helper, and catalyst components. The blue arrows point to bands corresponding to AR-H1 when this complex is produced as a result of undesired interactions between the Helper strand and ARL complex in the absence of Catalyst. The brown arrows points to the remaining ARL complexes when both Helper and Catalyst are present. The sequence names for each species, as listed in Supplementary Table 1, are: unlocked complex AR (AlHPCC_notT_noAc, RlHPCC_notT_noAc); locked complex ARL (AlHPCC_notT_noAc, RlHPCC_notT_noAc, locking strand *see below*); hairpin H_1 (H1); Helper (HelperlHPCC); Catalyst (CatalystlHPCC). The locking strands are GblHPCC (1 nt), GblHPCC2bp (2 nt), and GblHPCC3bp (3 nt). Experiment conducted as described in Supplementary Methods.

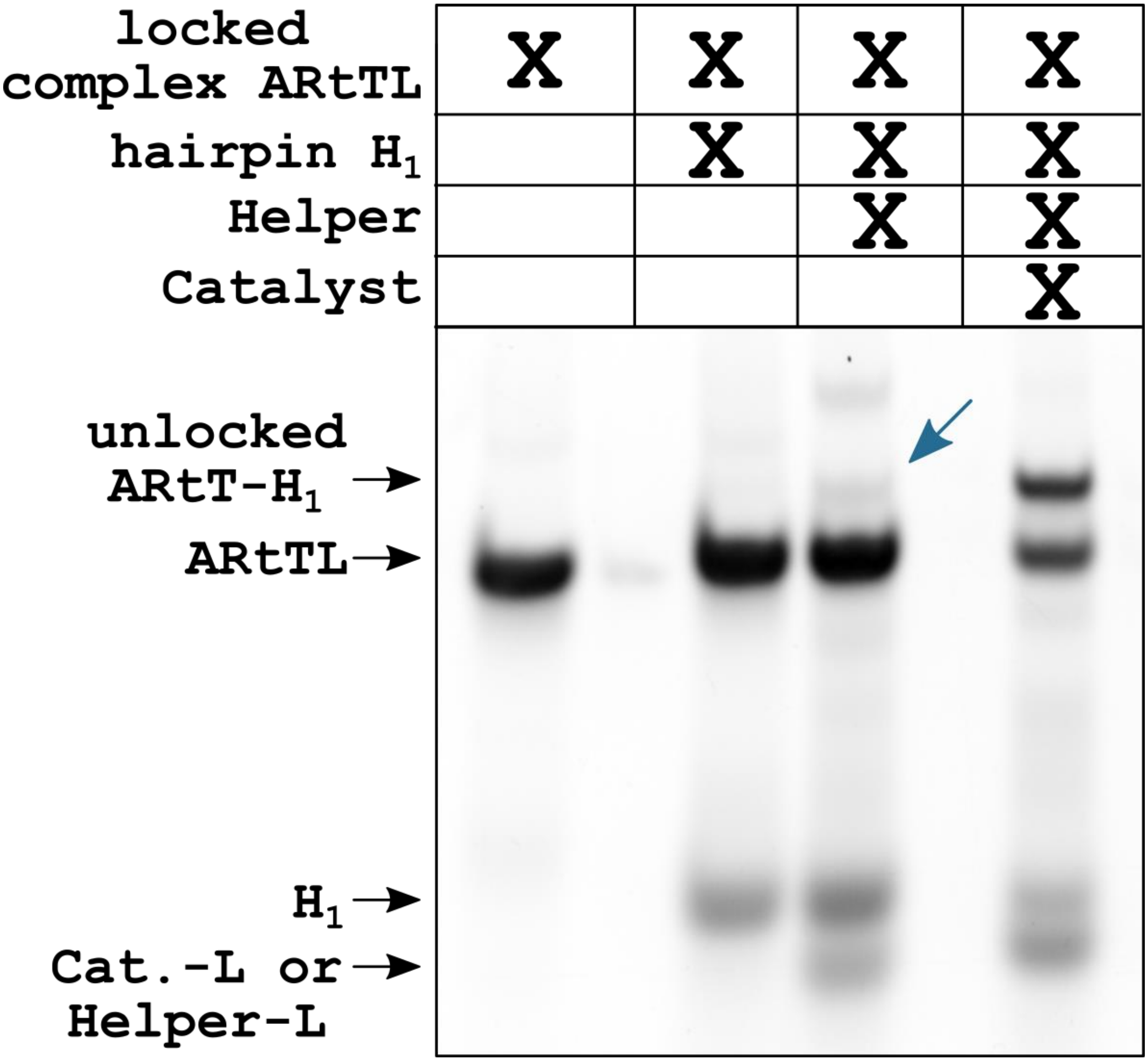

Supplementary Figure 6: PAGE gel demonstrating the effect of adding three thymine nucleotides to the 5’ terminus of the A strand and 3’ terminus of the R strand on the amount of unlocking and hairpin insertion in both the triggered and untriggered states. The blue arrow points to ARtT-H1 complex resulting from undesired interactions between the Helper strand and ARtTL complex in the absence of Catalyst. The sequence names for each species, as listed in Supplementary Table 1, are: locked complex ARtTL (AlHPCC_noAc, RlHPCC_noAc, GblHPCC); hairpin H_1 (H1); Helper (HelperlHPCC); Catalyst (CatalystlHPCC). Experiment conducted as described in Supplementary Methods.

Supplementary Table 1: List of sequences used in this study. Sequences were taken from either previous literature[2,3,5] or designed using NUPACK[1] as noted in Supplementary Methods and Supplementary Figs. 2, 9, 18, and 22. The crosslink strands *AlHPCC(v1)* and *RlHPCC(v1)* were used in main text Figure 2b and for measuring the swelling of the hydrogels prepared without locks (data labeled "no locks" in figures in main text and in supplementary information). The experiment for Supplementary Fig. 10 was conducted using *A1* and *R1* crosslinks. The crosslink strands *AlHPCC*, *RlHPCC*, *ASys2*, and *RSys2* were used for the particle swelling measurements presented in Supplementary Fig. 13.

Strand Name	Role	Sequence
**Crosslinks**		
A1	Original Crosslink	/5Acryd/TAAGTT CGCTGTGGCACCTGCACG
R1	Original Crosslink	/5Acryd/CAA CGTGCAGGTGCCACAGCG TGG
AlHPCC	Lockable Crosslink	/5Acryd/TT GA GTATTGT TAAGTT CGCTGTGGCACCTGCACG TTG
RlHPCC	Lockable Crosslink	/5Acryd/CAA CGTGCAGGTGCCACAGCG TGG GGTG TTT
AlHPCC (v1)	Crosslink (S.Fig. 4, "no locks" data)	/5Acryd/TGGT TAAGTT CGCTGTGGCACCTGCACG TTG
RlHPCC (v1)	Crosslink (S.Fig. 4, "no locks" data)	/5Acryd/CAA CGTGCAGGTGCCACAGCG TGG GG
ASys2	Sys. 2 Crosslink (S.Fig. 8)	/5Acryd/TT GT TATGTAT CTGTCT GCCTACCACTCCGTTGCG AAT
RSys2	Sys. 2 Crosslink (S.Fig. 8)	/5Acryd/ATT CGCAACGGAGTGGTAGGC TTT GAAT TTT
AlHPCC Sys2	Sys. 2 Lockable Crosslink	/5Acryd/TT GT TATGTAT CTGTCT GCCTACCACTCCGTTGCG AAT
RlHPCC Sys2	Sys. 2 Lockable Crosslink	/5Acryd/ATT CGCAACGGAGTGGTAGGC TTT GAAT TTT
**Locking/Unlocking Strands**		
GblHPCC	Locking Strand (1bp loop)	ATCT CACC CCA T AACTTA ACAATAC TC
FClHPCC	Key Strand	GA GTATTGT TAAGTT A TGG GGTG AGAT
HelperlHPCC	Helper Strand	GTATTGT TAAGTT TGG GG
CataylstlHPCC	Catalyst Strand	GT TAAGTT TGG GGTG AGAT
GblHPCC Sys2	Sys. 2 Locking Strand (1bp loop)	CACA ATTC AAA T AGACAG ATACATA AC
FClHPCC Sys2	Sys. 2 Key Strand	GT TATGTAT CTGTCT A TTT GAAT TGTG
HelperlHPCC Sys2	Sys. 2 Helper Strand	TATGTAT CTGTCT TTT GA
CataylstlHPCC Sys2	Sys. 2 Catalyst Strand	AT CTGTCT TTT GAAT TGTG

(continued on next page)

Hairpin Strands		
H1	Hairpin Monomer	CCA CGCTGTGGCACCTGCACG CACCCA CGTGCAGGTGCCACAGCG AACTTA
H2	Hairpin Monomer	TGGGTG CGTGCAGGTGCCACAGCG TAAGTT CGCTGTGGCACCTGCACG TTG
H1terminator	Hairpin Monomer	CCA CGCTGTGGCACCTGCACG TAGACT CGTGCAGGTGCCACAGCG AACTTA
H2terminator	Hairpin Monomer	TGGGTG CGTGCAGGTGCCACAGCG GCCTAG CGCTGTGGCACCTGCACG TTG
Q1	Sys. 2 Hairpin Monomer	AAA GCCTACCACTCCGTTGCG GAACCT CGCAACGGAGTGGTAGGC AGACAG
Q2	Sys. 2 Hairpin Monomer	AGGTTC CGCAACGGAGTGGTAGGC CTGTCT GCCTACCACTCCGTTGCG AAT
Q1terminator	Sys. 2 Hairpin Monomer	AAA GCCTACCACTCCGTTGCG TCAAGC CGCAACGGAGTGGTAGGC AGACAG
Q2terminator	Sys. 2 Hairpin Monomer	AGGTTC CGCAACGGAGTGGTAGGC AATCGT GCCTACCACTCCGTTGCG AAT
**Logic Converter**		
Eo7Cat	Logic Gate	GTTAGATG G AGAT GT AATTGATATG T GT GAG G AATGAT
GbEFG	Logic Gate	GTTCCCTGATCTTTA GCCTTA ATCATT C CTC AC A AC ATCT C CATCTAAC
G	Logic Gate	TAAGGC TAAAGATCAGGGAAC ACCATA
G.in	Logic Input	TATGGT GTTCCCTGATCTTTA GCCTTA
Fcatst.in	Logic Input/Purif.	GTTAGATG G AGAT GT T GT GAG G AATGAT TAAGGC
G.in.NoToe	Logic Gate Purification	GTTCCCTGATCTTTAGCCTTA
Wcatalyst_7	Logic-Cat. Converter	GT TAAGTT TGG GGTG AGAT GT AATTGATATGT GT
GbC7	Logic-Cat. Converter	AC CTC AC ACATATCAATT AC ATCT C
**ATP Sensor/Converter**		
Cof.tapt_eta	Cofactor	TGAGG GT AGTGGAGTGAG G
Weta_ATPapt	ATPSensor Gate	GT AGTGGAGTGAG GT GAG G ACCTGGGGGAGTATTGCGGAGGAAGGT
Gbeta_ATPapt	ATPSensor Gate	CCAGGT C CTC AC CTCACTCCACT AC CCTCA
Wcatalyst_eta	Aptamer-Cat. Converter	GT TAAGTT TGG GGTG AGAT GT AGTGGAGTGAG GT
GbCeta	Aptamer-Cat. Converter	AC CTC AC CTCACTCCACT AC ATCT C
**Controller Reporting Assay Strands**		
Rv(Wcat)q	Reporter	/5IABkFQ/GT TAAGTT TGG GG TG
Rb(Wcat)f	Reporter	C ATCT CA CC CCA AACTTA AC/36-FAM/
PolyT20	polyT	TTTTTTTTTTTTTTTTTTTT

(continued on next page)

Extra PAGE Analysis Strands		
HelperNTlHPCC	HelperNT Strand (not truncated)	GTATTGT TAAGTT TGG GG T
GblHPCCnl	Locking Strand (no loop)	ATCT CACC CCA AACTTA ACAATAC TC
GblHPCC2bp	Locking (2bp loop) Strand	ATCT CACC CCA CT AACTTA ACAATAC TC
GblHPCC3bp	Locking Strand (3bp loop)	ATCT CACC CCA CTT AACTTA ACAATAC TC
AlHPCC_notT_noAc	Lockable Complex (no acrydite/polyT domain)	GA GTATTGT TAAGTT CGCTGTGGCACCTGCACG TTG
RlHPCC_notT_noAc	Lockable Complex (no acrydite/polyT domain)	CAA CGTGCAGGTGCCACAGCG TGG GGTG
AlHPCC_noAc	Lockable Complex (no acrydite)	TTT GA GTATTGT TAAGTT CGCTGTGGCACCTGCACG TTG
RlHPCC_noAc	Lockable Complex (no acrydite)	CAA CGTGCAGGTGCCACAGCG TGG GGTG TTT

Supplementary Note 2: Measuring the area of a particle's 2D fluorescence projection

The area of the 2D projection of each particle in the fluorescence micrographs (Supplementary Fig. 7) was calculated using custom written MATLAB scripts developed using standard edge-detection algorithms. The algorithm used thresholding to determine the boundaries of the particles. This threshold value was calculated using the following method:

1. Normalize the image to the highest and lowest intensity.

$$\text{normImage} = \frac{\text{Image} - \text{min(Image)}}{\text{max(Image)} - \text{min(Image)}} \tag{1}$$

2. Use MATLAB's built-in global threshold calculator *graythresh.*

$$\text{globalThresh} = \mathit{graythresh}(\text{normImage}) \tag{2}$$

3. Convert the normalized global threshold into an absolute global threshold and perform image-specific adjustments.

$$\text{Thresh} = \text{globalThresh} * (\mathit{max}(\text{Image}) - \mathit{min}(\text{Image})) + \mathit{min}(\text{Image}) \tag{3}$$

$$\text{intThreshOrig} = \text{Thresh of 1st image in time series} \tag{4}$$

$$\beta = \frac{\mathit{mean}(\text{Image}) * \alpha}{\text{intThreshOrig}} \tag{5}$$

$$1.1 \leq \alpha \leq 3 \tag{6}$$

$$\text{particlePixels} = \text{Image} > \beta * \text{Thresh} \tag{7}$$

where α corresponds to a manual input that was adjusted until a close match between the visible particle boundaries and the calculated boundaries was found. By converting the normalized global threshold (which can be used to threshold the normalized image) into the absolute global threshold, the values chosen for α are more standard between image sets and our measurements are more robust against irregularly bright pixels. *particlePixels* is a logical image mask where values corresponding to 1 indicate pixels that are within the particle's boundary. For

particles that did not have significant intensity changes over the course of the experiment (*i.e.*, particles that did not swell to a significant extent), α did not need to be adjusted between images in a time series. For some particles that did swell, and thus did have significant intensity changes, α was manually adjusted about every 5 images. After determining the pixels corresponding to the particle, the particle's area and boundary were extracted using the functions *regionprops* and *bwboundaries*. Examples of processed images are shown in Supplementary Fig. 7.

The area as determined by *regionprops* was converted into square micrometers using the image's pixel size (4.44 μm/pixel) and the relative change in area as a function of time was calculated using:

$$\Delta\text{area}(t) = \frac{\text{area}(t) - \text{area}(t = 0)}{\text{area}(t = 0)} \cdot 100\% \tag{8}$$

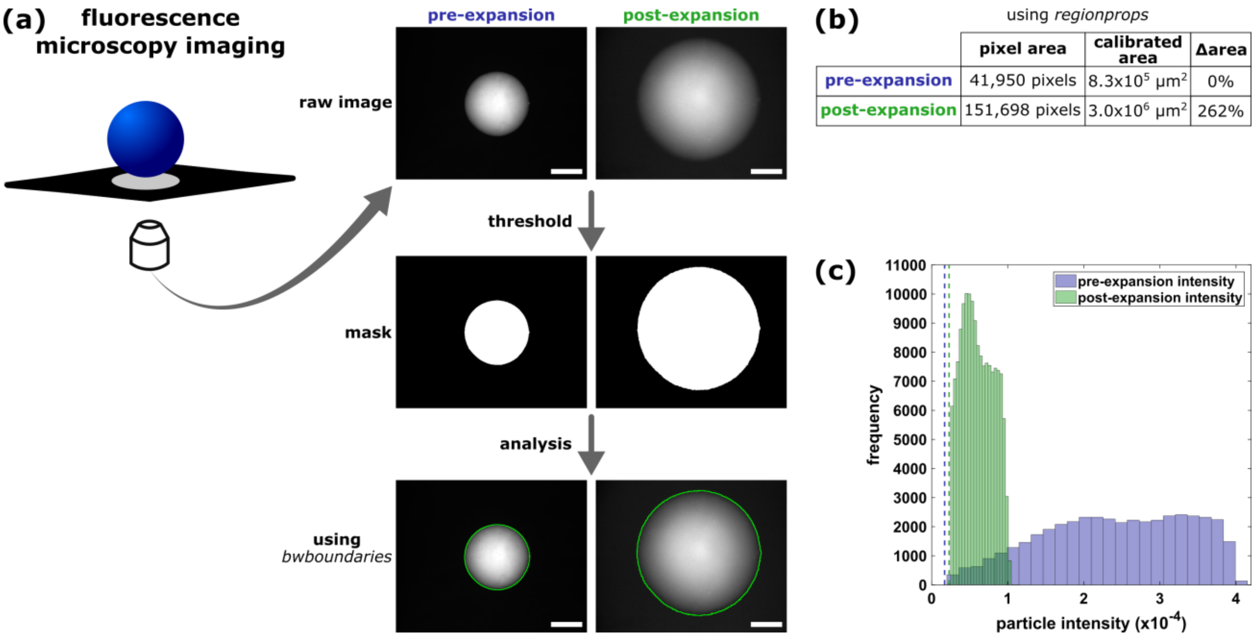

Supplementary Figure 7: Measuring the area and intensity of a particle's 2D fluorescence projection. (a) Particles were imaged using fluorescence microscopy and the area of the particle as seen in the 2D micrograph was determined using MATLAB (Supplementary Note 2). The white pixels in the middle set of images represent the set of pixels determined to belong to the particle after image thresholding. The green line around each particle in the bottom images represent the calculated boundary of each particle. Image intensities are scaled based on each image's minimum and maximum intensity. Scale bars: 500 µm. (b) The area of the pixels within the green boundary is the calculated particle projection area and was calculated using the MATLAB function *regionprops*. The number of pixels is converted to square microns and the relative change in area. (c) Histogram of the intensity of the particles (pixels within the green boundary) shown in (a). As the particle expands, the intensity of the particle decreases because the density of rhodamine fluorophores decreases. Dashed lines indicate the average background intensity of each image.

Supplementary Note 3: Calculating particle radii and circularity

The radius of a particle was determined by calculating the average distance between each of the points on the particle's boundary, as determined by the methods described in Supplementary Note 2, and the centroid of the particle. Particle centroids were determined using the function *regionprops* and the distance in pixels was calculated using the function *pdist2* in MATLAB. The average radius in pixels was converted to microns by multiplying the radius by the image's pixel size (4.44 micrometers/pixel).

The circularity of each particle was calculated using the area of the particle and the perimeter of the particle boundary, both calculated using the *regionprops* function in MATLAB:

$$\text{Circularity} = \frac{4\pi \cdot \text{Area}}{\text{Perimeter}^2} \tag{9}$$

A score of 1 indicates a perfect circle.

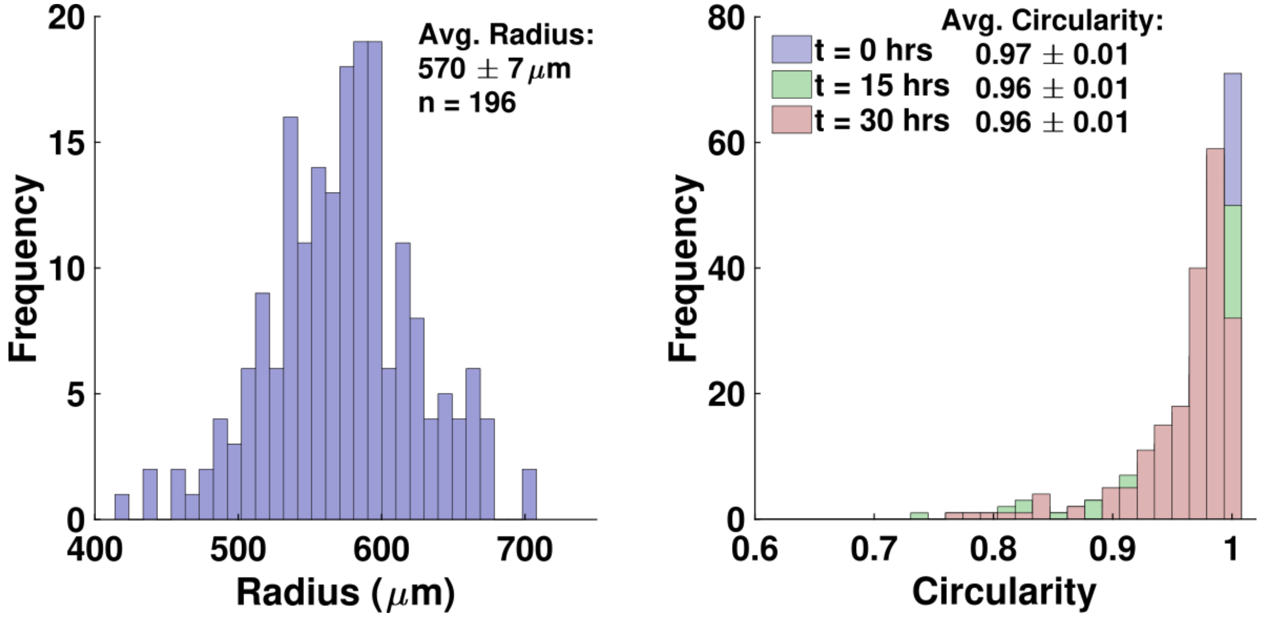

Supplementary Figure 8: Average radii and circularity of DNA-crosslinked hydrogel particles. (a) Distribution of rhodamine-labeled poly(DNA-*co*-acrylamide) particle radii prior to expansion determined as described in Supplementary Note 3. The uncertainty in the average radius is the 95% confidence interval calculated from the standard deviation. (b) Circularity scores of particles (N=196), determined as described in Supplementary Note 3. A particle with a circularity score of 1 is a perfect circle. The mean circularity is 0.97±0.01 at time 0 and 0.96±0.01 after 30 hours of incubation with hairpins. These statistics are derived from the particles used in the experiments in this study, so particles that expand to different extents are included in these time averages. Uncertainties are the 95% confidence intervals calculated from the standard deviations.

Supplementary Note 4: Weight fraction and mole fraction calculations

Since the reaction systems for hydrogel swelling is biphasic, with one phase being the hydrogel and the other the surrounding liquid, the total mass of each species is important for understanding the behavior of the system. We calculated the mass of each species in the pre-gel solution and in a hydrogel expanded using DNA hairpins (Supplementary Table 2). We assumed that each species in the pre-gel solution was fully polymerized into the hydrogel, that the volume of the pre-gel solution was 0.25 µL, and that all of the DNA hairpins were incorporated into the hydrogel. The efficiency of acrydite-modified DNA incorporation onto solid supports was previously measured to be around 83%, albeit using different polymerization initiating agents than used here.[11] Water weight was not included in the mass calculations. The calculations show that if all of the hairpins are incorporated into the hydrogel, the weight fraction of polyacrylamide decreases by a factor of ~3 and the total amount of DNA increases 5-fold.

Supplementary Table 2: Calculations of the ideal mass and weight fraction of each species inside the pre-gel solution and the expanded hydrogel. Calculations assume each monomer species becomes polymerized into the gel from the pre-gel solution and that all hairpins are incorporated into the expanded gel.

		pre-gel			expanded gel		
species	MW (g/mol)	conc. (mM)	mass (µg)	weight fract. (w/w%)	conc. (mM)	mass (µg)	weight fract. (w/w%)
DNA crosslink	14810.6	1.154	4.27	14.37	-	4.27	4.64
acrylamide	71.08	1407	25.00	84.09	-	25.00	27.17
rhod.-methacryl.	666.2	2.74	0.46	1.53	-	0.46	0.50
hairpins	15576.1	0	0	0	0.04	62.30	67.70

We also calculated the mole fraction of the DNA species present under triggerable expansion conditions, *i.e.*, when converting locked crosslinks to an unlocked state (Supplementary Table 3). Under these reaction conditions, the hydrogel is but a fraction of the total volume. Using the average radius of the particles (Supplementary Figure 8), the average particle volume prior to adding hairpins is 0.8 mm^3, about 0.8% of the total volume (~100 µL). These calculations also assume that all of the DNA crosslinks are polymerized into the hydrogel. Based on the calculations, at most about 14 hairpins would be incorporated at each crosslink site. Terminating hairpins would decrease this number. However, 14 hairpins increase the crosslink length from about 7.5 nm to 111 nm, a length increase of about 15-fold.

The relative mole fraction of Key strand in the unlocking experiments is consistent with the hypothesis that only a fraction of crosslinks become unlocked with 1 µM or lower Key strand, but most, if not all, crosslinks should become unlocked at the high concentration of 10 µM Key strand. The number of moles of Catalyst strand, however, is lower than that of the DNA crosslinks, indicating that catalytic turnover is indeed necessary to induce the high-degree of swelling measured for 75-100 nM Catalyst strand.

Supplementary Table 3: Number of moles of each species in triggerable expansion conditions. Calculations assume all DNA crosslinks are polymerized into the hydrogel.

species	conc. (mM)	vol. (µL)	nmole species	mol species/mol crosslink
DNA crosslinks	1.154	0.25	0.2885	1
hairpins	0.04	100	4	13.9
Key	0.01	100	1	3.5
Helper	0.01	100	1	3.5
Catalyst	0.0001	100	0.01	0.035

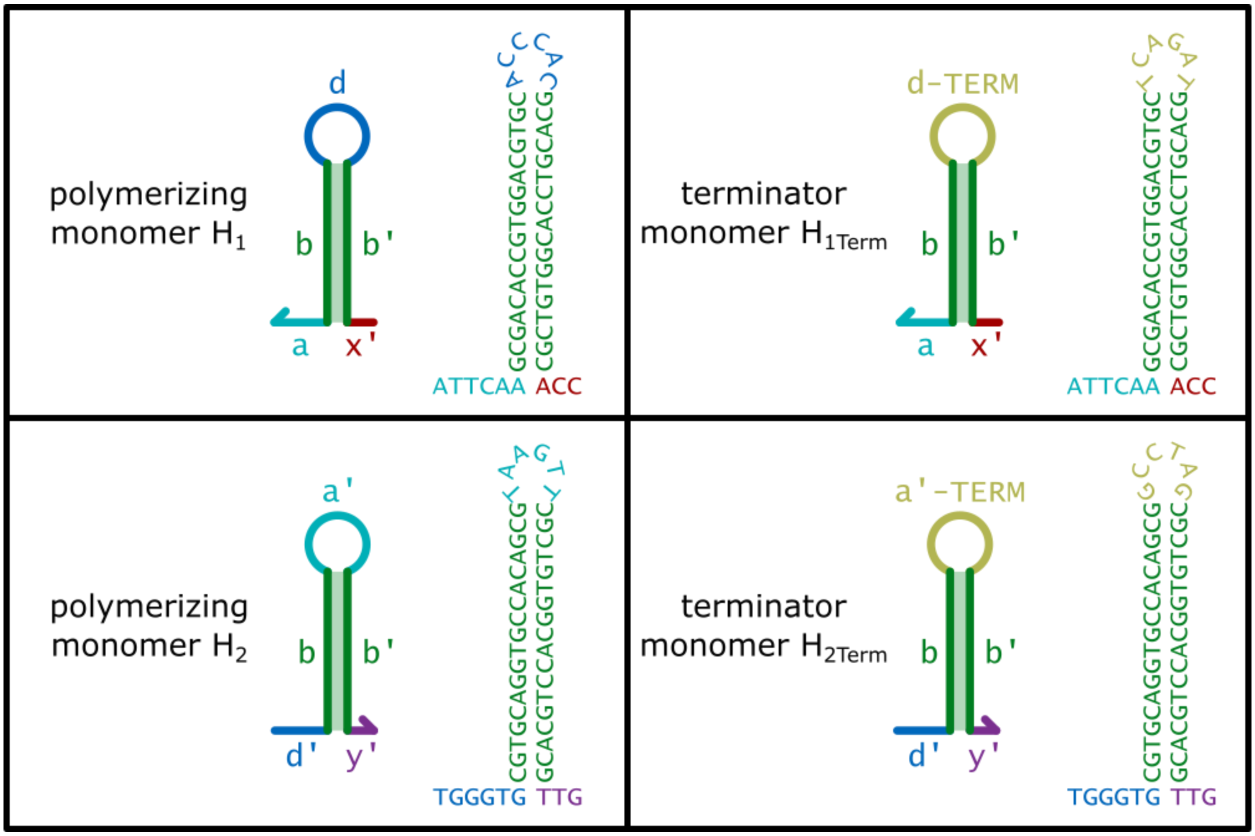

Supplementary Figure 9: DNA hairpin monomer sequences.[2] Terminator monomers share the same sequence as their polymerizing monomer counterpart except for the loop region so that after insertion of a terminator monomer, other hairpin monomers cannot insert into the crosslink.

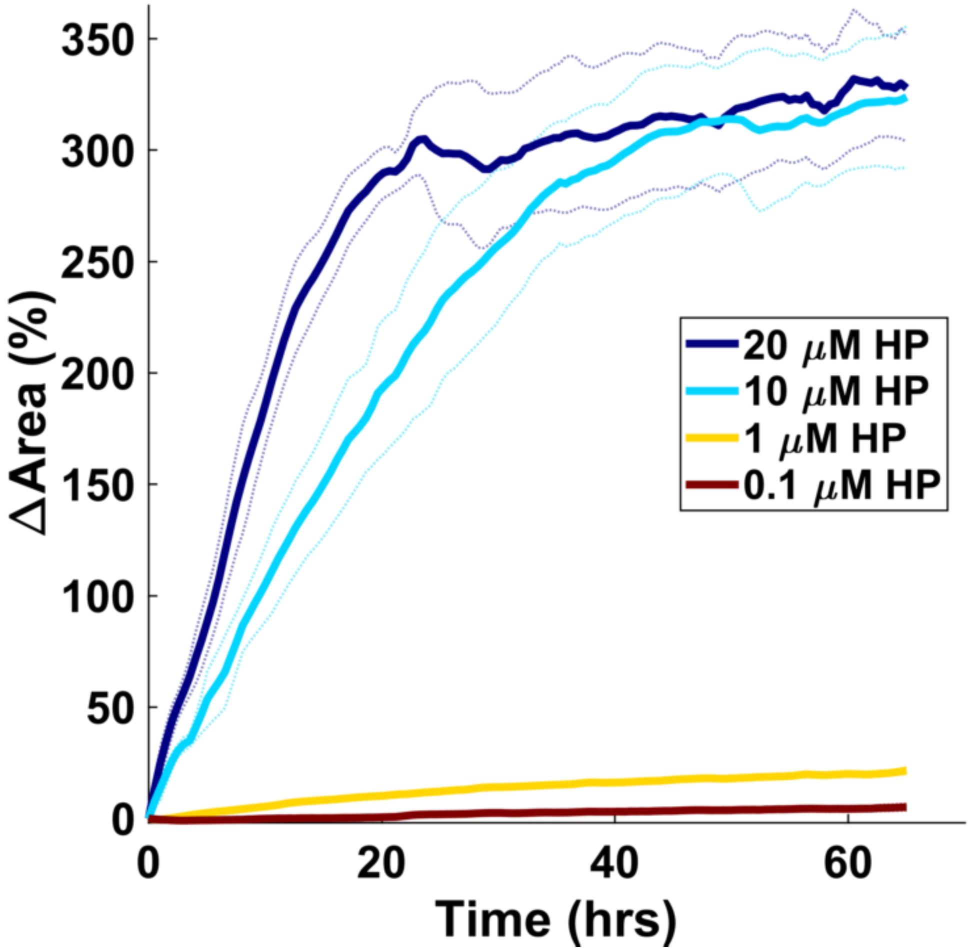

Supplementary Figure 10: Particles prepared without crosslink locks incubated with different concentrations of hairpin monomers (concentration per type is shown). In all cases, the percentage of terminator monomer is 10%. Solid curves are the averages of measurements of 2 particles (replicate measurements shown as dotted lines).

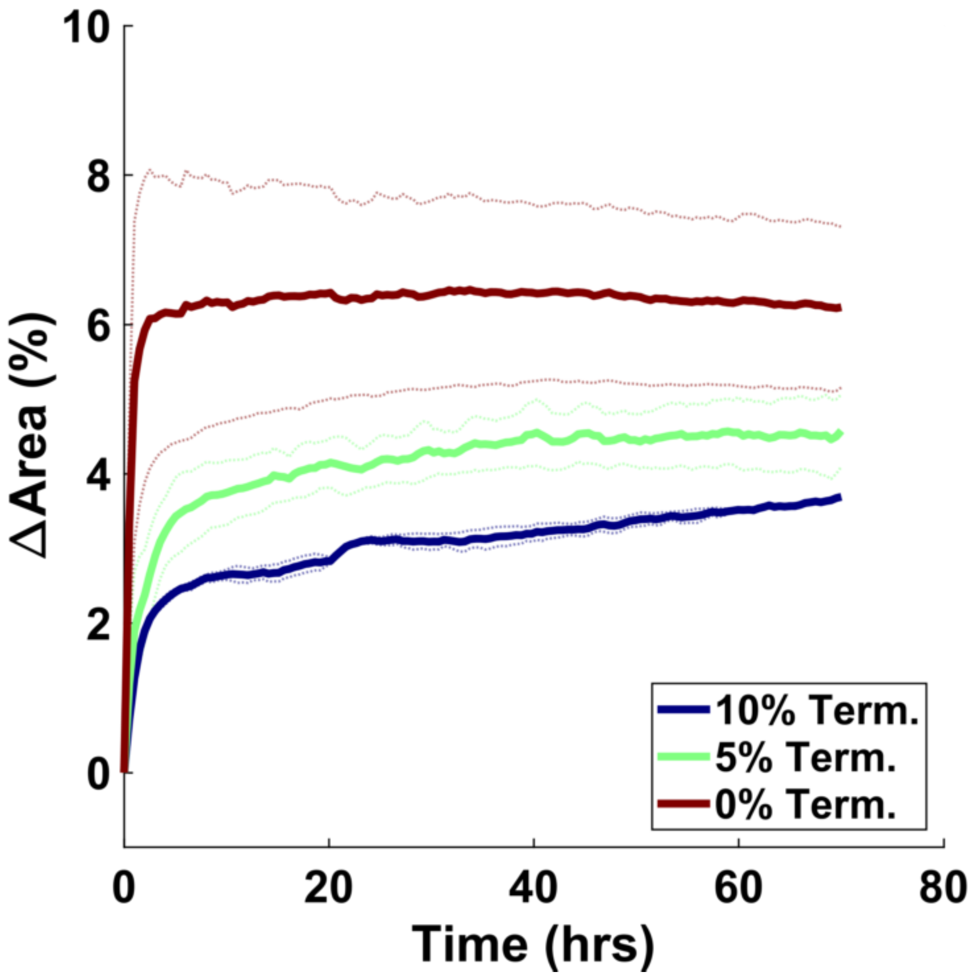

Supplementary Figure 11: Locked crosslink hydrogel particles show 50-fold less swelling than hydrogels with no locks. Swelling was tested with different percentages of terminator monomer over 70 hours and 20 μM per hairpin monomer type. Solid curves are the averages of measurements of two particles (replicate measurements shown as dotted lines).

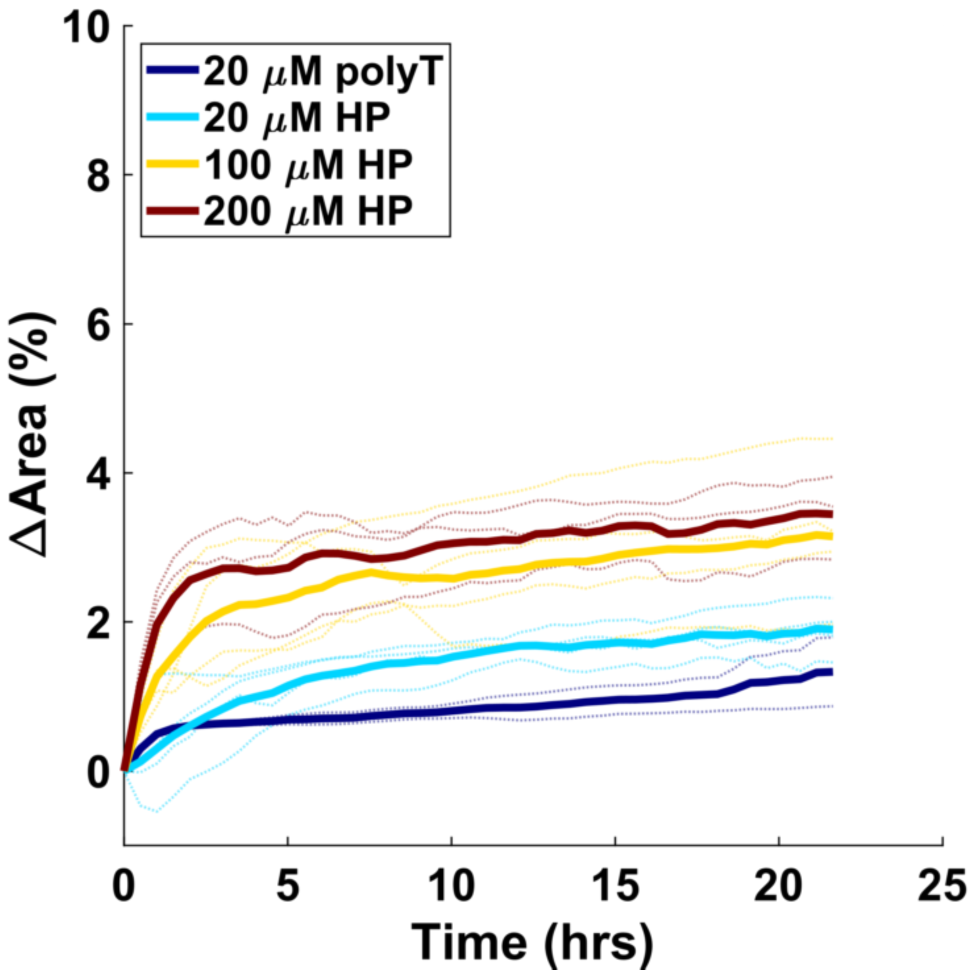

Supplementary Figure 12: Swelling of locked particles incubated with different concentrations of hairpin monomers or a 20-mer of thymine (polyT). In all cases with hairpins, the fraction of terminator monomer is 10%. Curves are the averages of measurements of 2 (polyT), 3 (200 µM), or 4 (20 µM, 100 µM) particles. Dotted lines are the replicate measurements for each average.

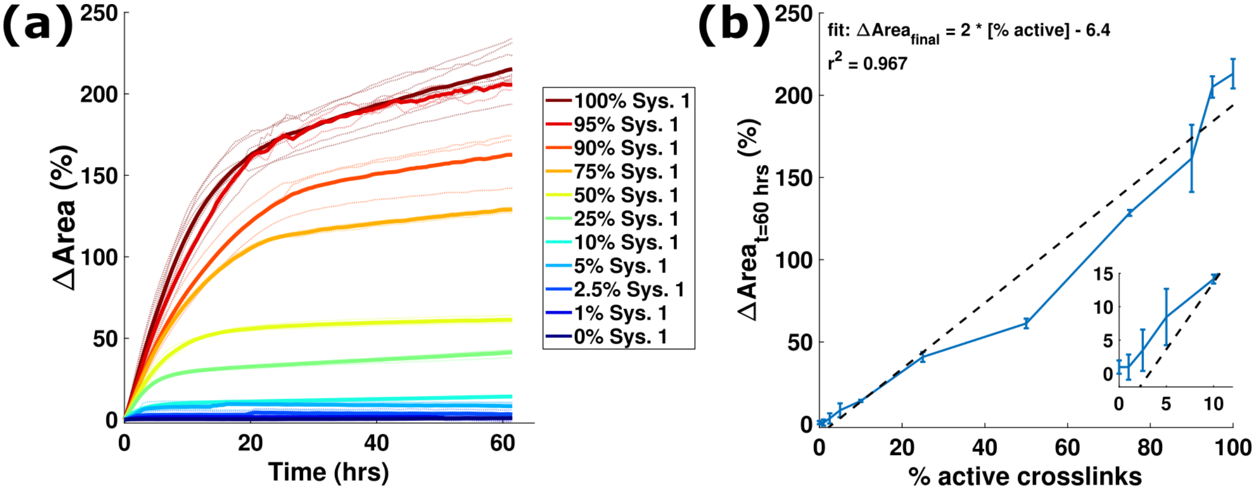

Supplementary Figure 13: Swelling kinetics of particles prepared with different fractions of expandable crosslinks (System 1) *vs*. crosslinks with different sequences designed to not interact with the Key strand and hairpin monomers (System 2, sequences in Supplementary Table 1). (a) Particles were incubated with 20 µM hairpin monomers of which 10% were terminator monomers. The legend gives the percentage of crosslinks that are expandable *via* hairpin monomers. Hydrogels with 0% System 1 contain 100% System 2 crosslinks. The lack of response of these gels to System 1 hairpins demonstrates the sequence specificity of the swelling process, consistent with previous studies.[2] The curve for 100% System 1 crosslinks shows the average expansion of measurements of 8 particles. All other curves show the averages of measurements of 3 particles. Solid lines: averages of measurements; dotted lines: replicate measurements for each average curve. (b) The change in area at 60 hours of incubation with hairpin monomers as a function of the percentage of expandable (active) crosslinks. The dashed line is a linear fit to the measurements. Inset is a zoomed in portion of the plot showing the change in area for particles with 10% or less active crosslinks. Error bars: 95% confidence intervals as determined by standard deviations; replicate numbers listed in (a).

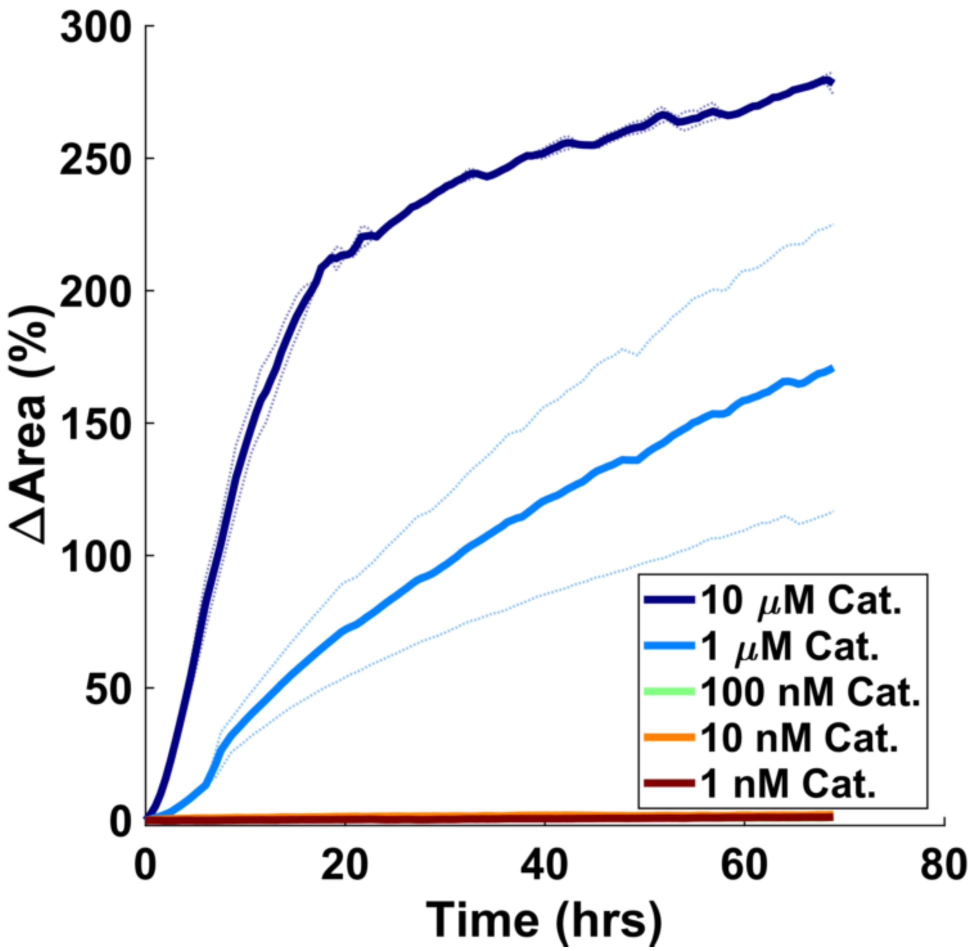

Supplementary Figure 14: Swelling of locked particles with hairpin monomers and different concentrations of Catalyst strand. The rate of swelling increases with increasing Catalyst concentration. Hairpin monomers are 20 μM per type with 10% terminator. Curves for 1, 10, and 100 nM Catalyst are indistinguishable from one another. Solid lines are the averages of measurements of two particles. Replicates for each average are shown as dotted lines.

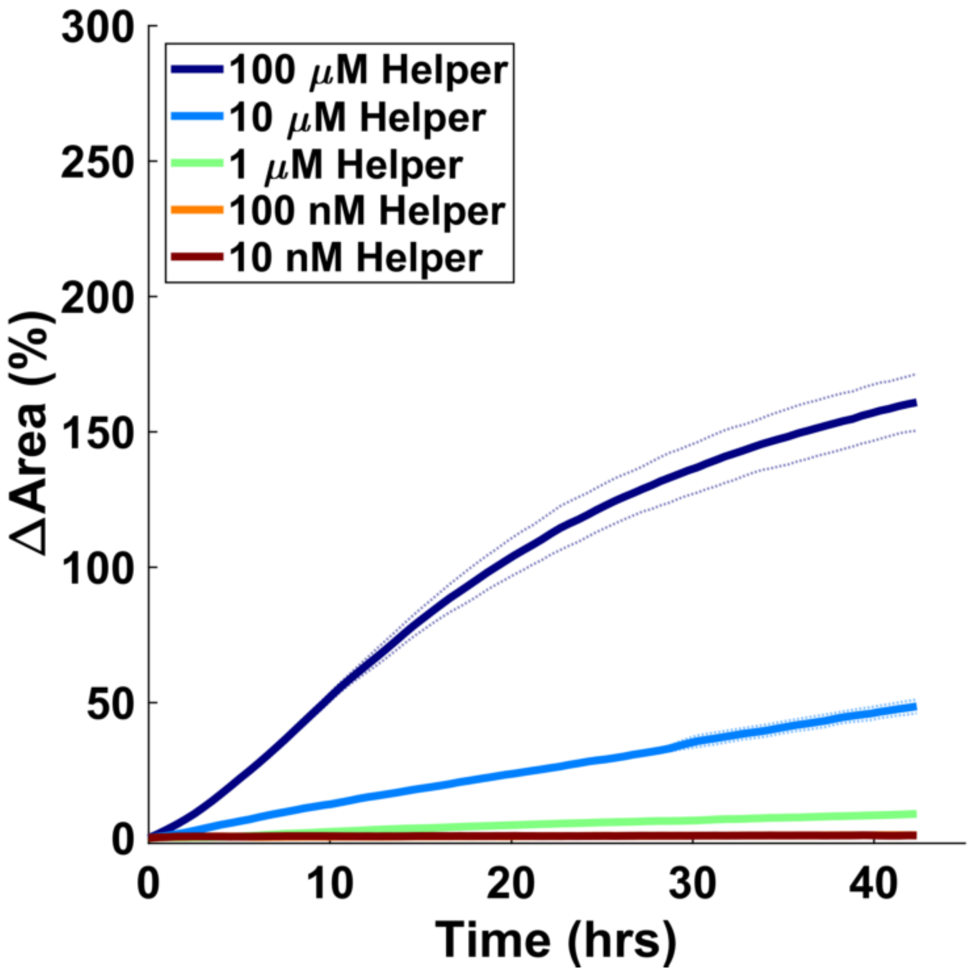

Supplementary Figure 15: Swelling of locked particles with hairpin monomers and different concentrations of Helper strand. Hairpin monomers are 20 µM per type with 10% terminator. Solid lines are the averages of measurements of two particles. Replicates for each average are shown as dotted lines.

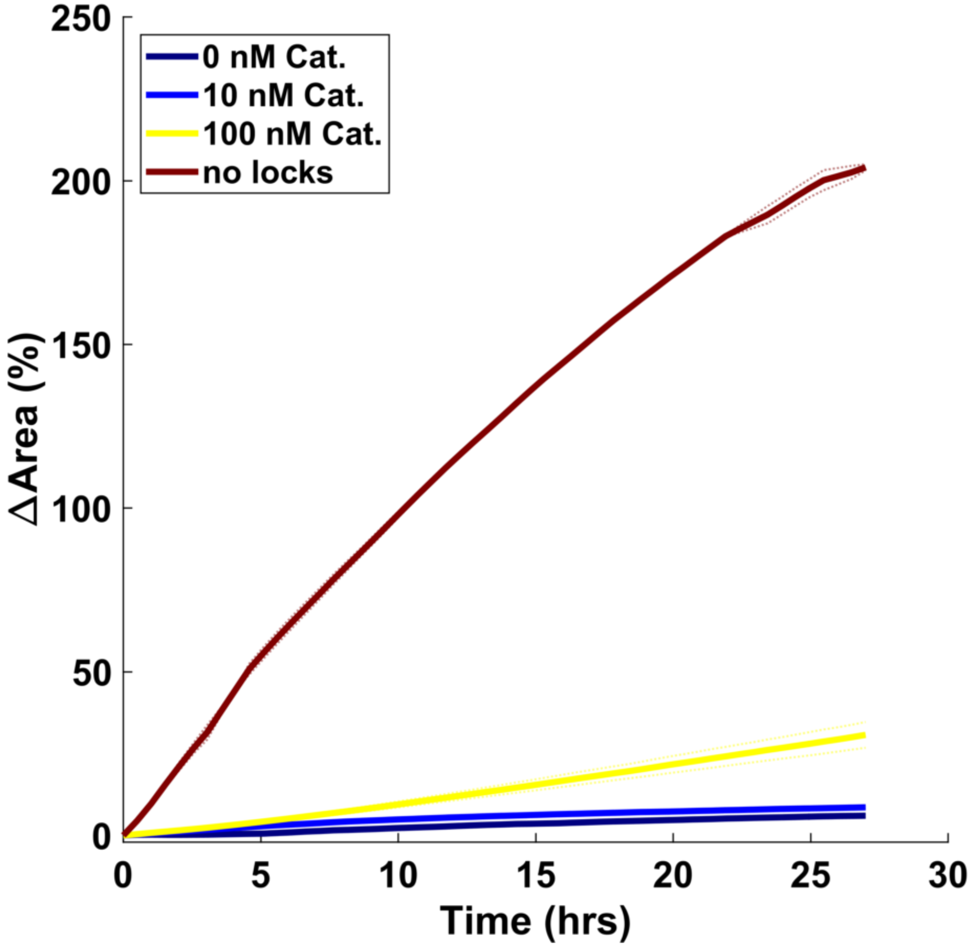

Supplementary Figure 16: Swelling rates of locked hydrogel particles in the presence of hairpin monomers, 1 μM Helper, and different concentrations of Catalyst. The amount of swelling observed over 25 hours is much less than the swelling observed when 10 μM Helper is present (Supplementary Fig. 17), indicating that a higher Helper concentration is needed for high catalytic turnover and high-degrees of swelling. The swelling of particles prepared without locks is shown for comparison. The hairpin monomer concentration for all curves is 20 μM per type with 10% terminator. Solid lines are the averages of measurements of 2 particles. Replicates for each average are shown as dotted lines.

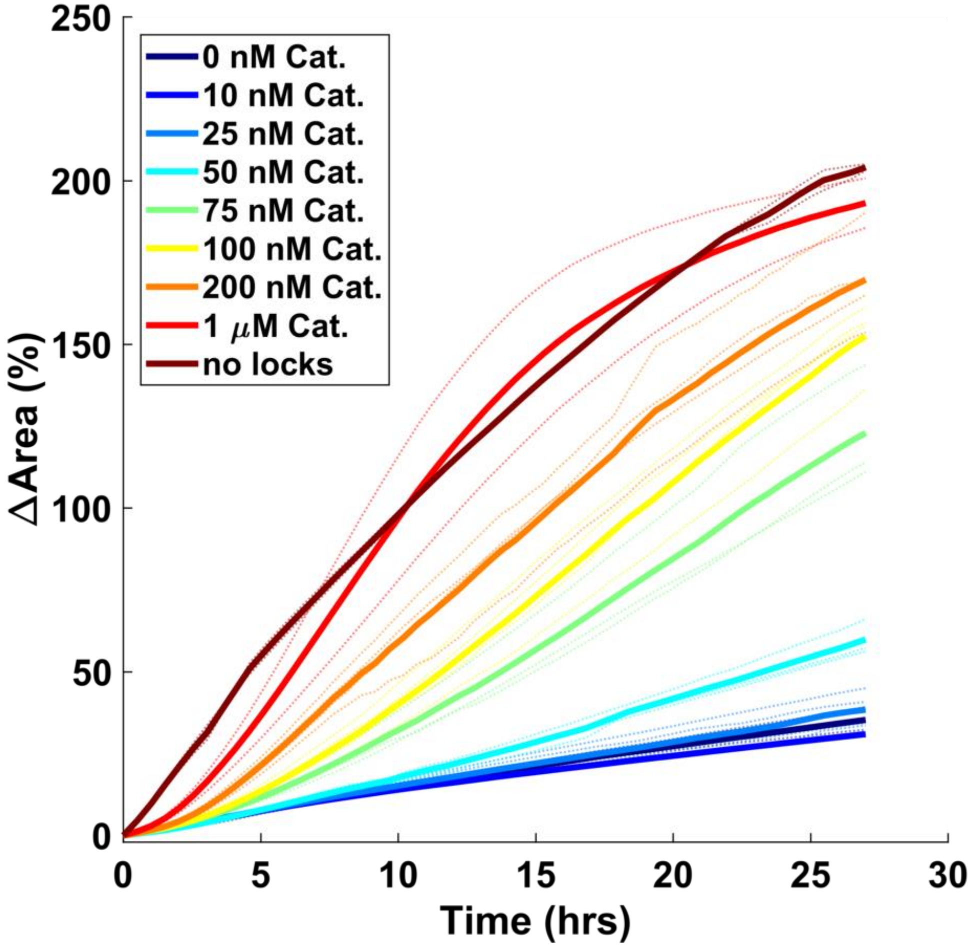

Supplementary Figure 17: Swelling over time for locked crosslink particles in the presence of 10 µM Helper, 20 µM of hairpins 1 and 2, of which 10% were terminator, and different concentrations of Catalyst. The swelling of particles prepared without locks is shown for comparison. Solid lines are the averages of measurements from 2 (10 nM, 1 µM, no locks), 3 (0 nM, 50 nM, 75 nM), or 4 (25 nM, 100 nM, 200 nM) particles. Replicates for each average are shown as dotted lines.

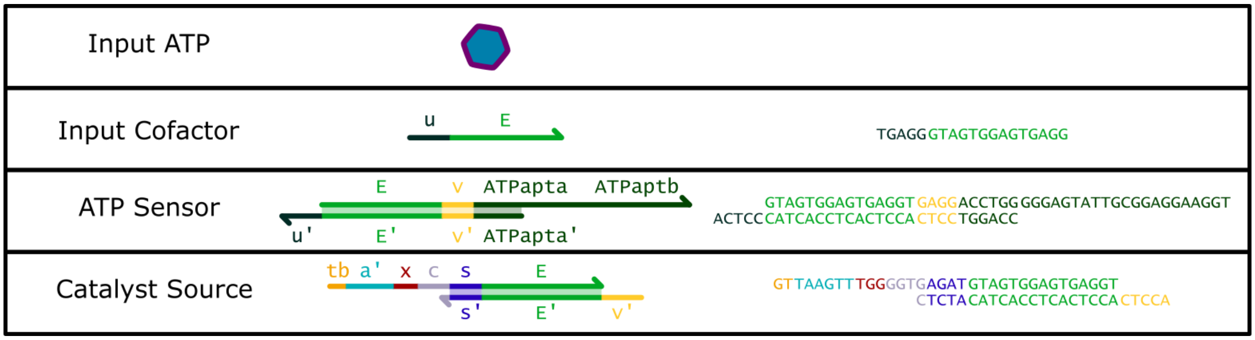

Supplementary Figure 18: Components of the ATP aptasensor circuit. *ATPapta* and *ATPaptb* (dark green domain) are the domains containing the ATP aptamer sequence.[4] The toeholds *u* and *v* and domain *E* were designed to have minimal nonspecific crosstalk with all other sequences using NUPACK (Supplementary Methods).[1]

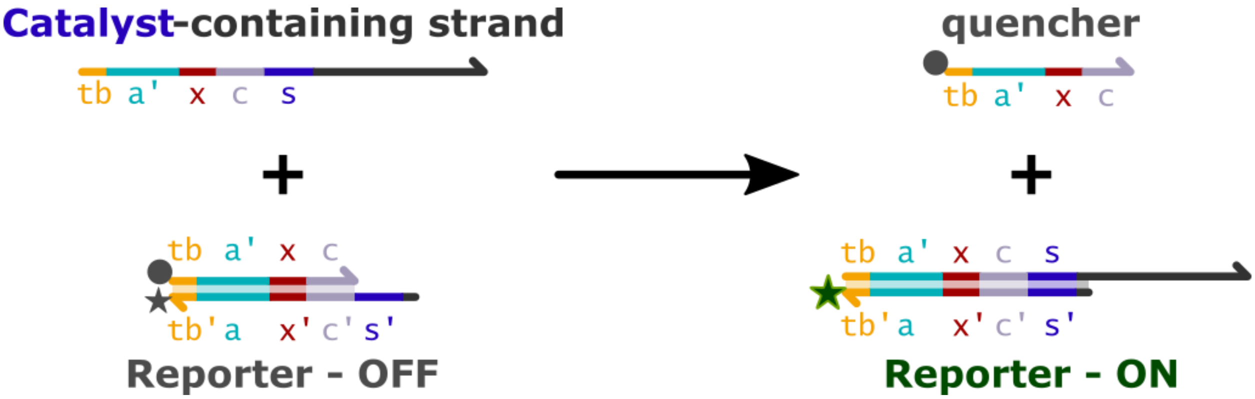

Supplementary Figure 19: Reporter reaction that detects the presence of exposed Catalyst sequence through an increase in fluorescence. Star is the FAM fluorophore, circle the IowaBlack quencher. The toehold on the Reporter is 5 bases (see Supplementary Table 1 for sequences).

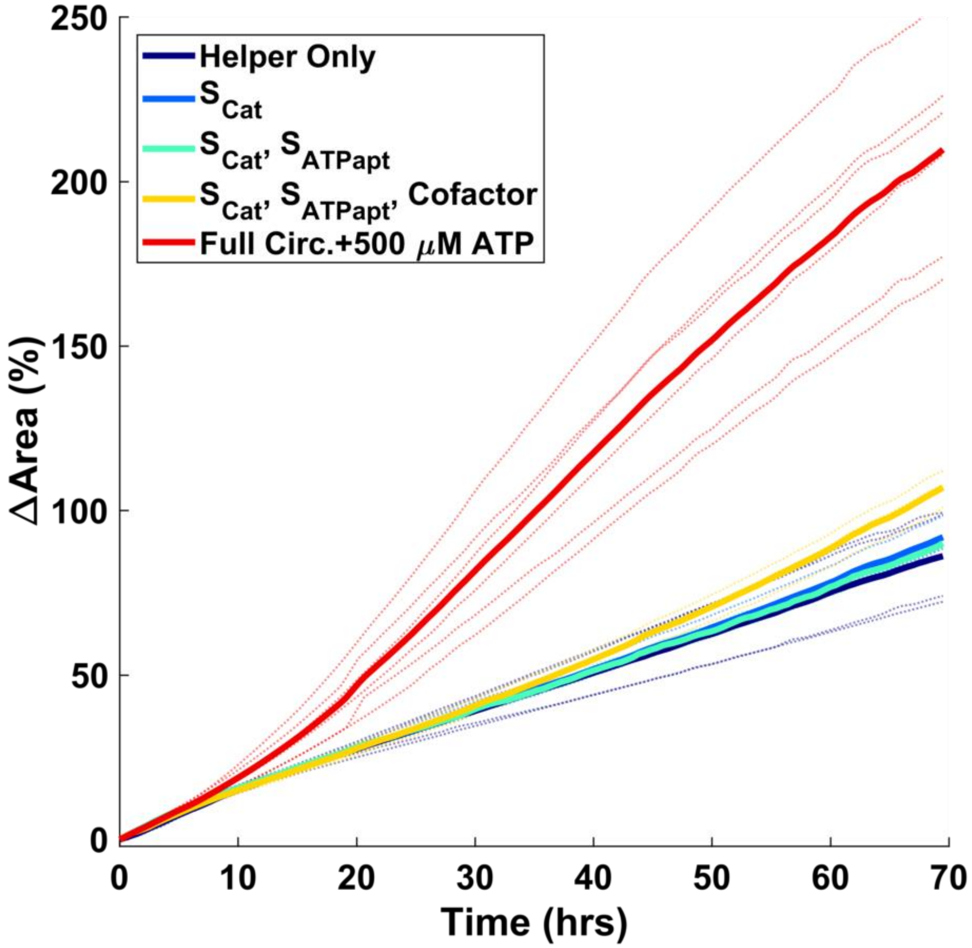

Supplementary Figure 20: Particles incubated with ATP aptasensor circuit components (Supplementary Figure 18), Helper strands, and hairpins do not swell significantly more than particles incubated with just Helper strands and hairpin monomers. Particles incubated with Catalyst Source and ATP Sensor complexes (S_{Cat} and S_{ATPapt}) at 100 nM did not show more swelling than particles containing only 10 µM Helper strand. Slightly more swelling is observed when the Cofactor is included at 100 nM than when no circuit components are included. The swelling of particles in the presence of the full circuit and 500 µM ATP is shown for comparison. Solid lines are the averages of measurements of 3 (S_{Cat}, S_{Cat}/S_{ATPapt}, S_{Cat}/S_{ATPapt}/Cofactor), 4 (Helper only), or 6 (Full circ./500 µM ATP) particles. Replicates for each average are shown as dotted lines.

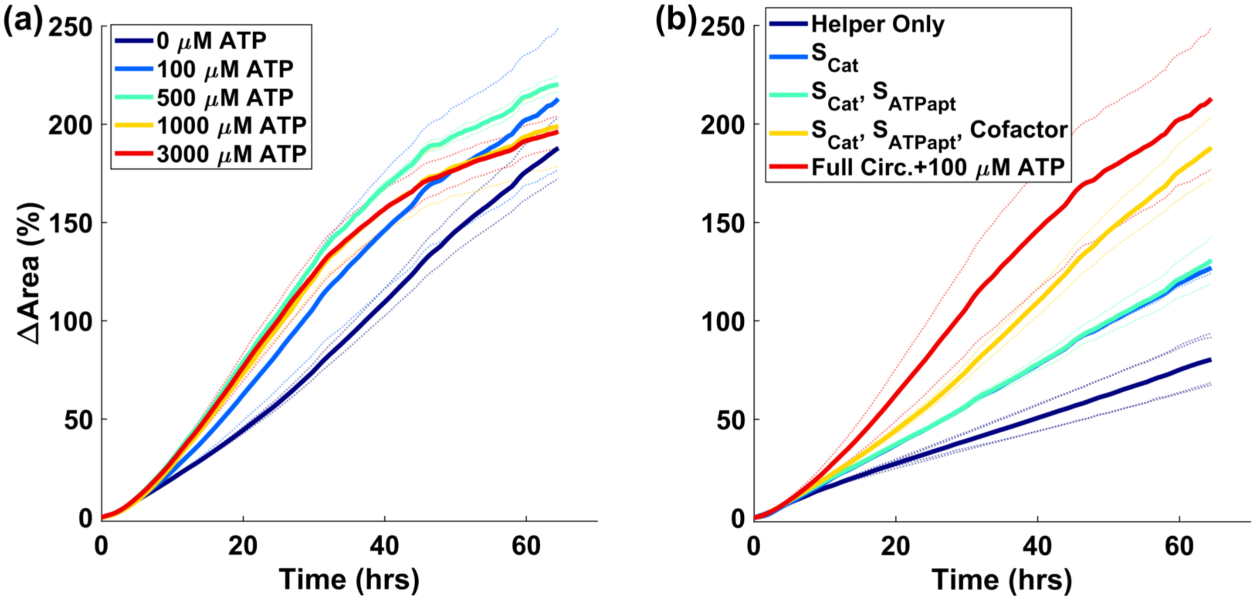

Supplementary Figure 21: (a) Increasing the concentration of the aptasensor circuit components (Catalyst Source, ATP Sensor, and Cofactor strand) to 200 nM from 100 nM (Supplementary Fig. 20) decreases the sensitivity of the swelling process to ATP concentration, perhaps because at higher concentrations circuit components can react with one another *via* unintended pathways at faster rates and thus produce more swelling in the off state. Curves are the averages of measurements of 2 particles. Dotted lines are replicate measurements used to calculate each average. (b) The presence of Catalyst Source and ATP Sensor complexes (S_{Cat} and S_{ATPapt}) or Cofactor at 200 nM increases the rate of swelling over the amount of observed in the presence of the Helper strand alone, even in the absence of ATP. Such an increase was not seen in the presence of 100 nM of the same circuit components (Supplementary Fig. 20). Solid lines are the averages of measurements of 4 (Helper only) or 2 particles. Dotted lines are replicate measurements used to calculate each average.

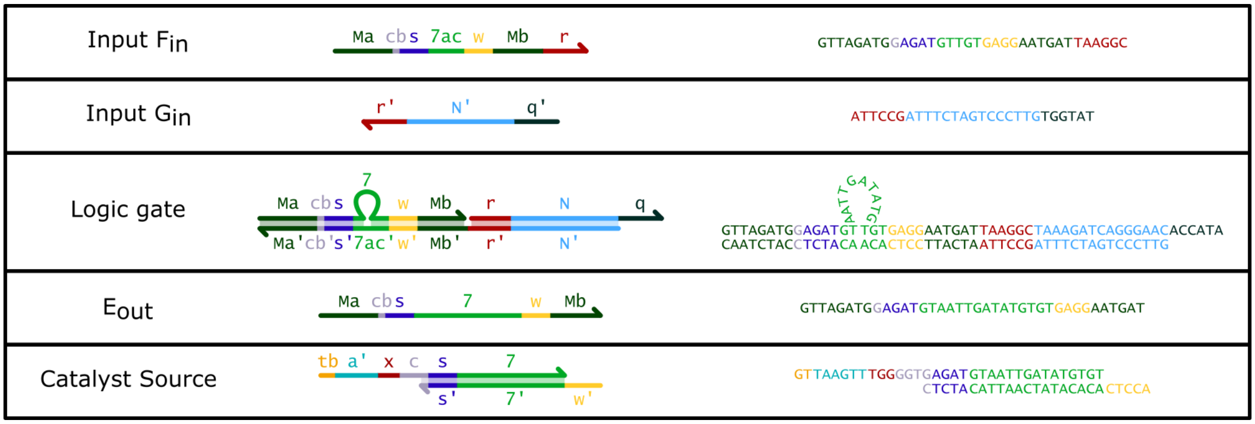

Supplementary Figure 22: Schematic of the components of the Logic Circuit. The circuit is based on a design from Seelig *et al*.[3] The toehold, *w*, was designed to have minimal nonspecific crosstalk with all other sequences using NUPACK (Supplemental Methods).[1] The sequence for domain *7* is from Qian and Winfree.[5]

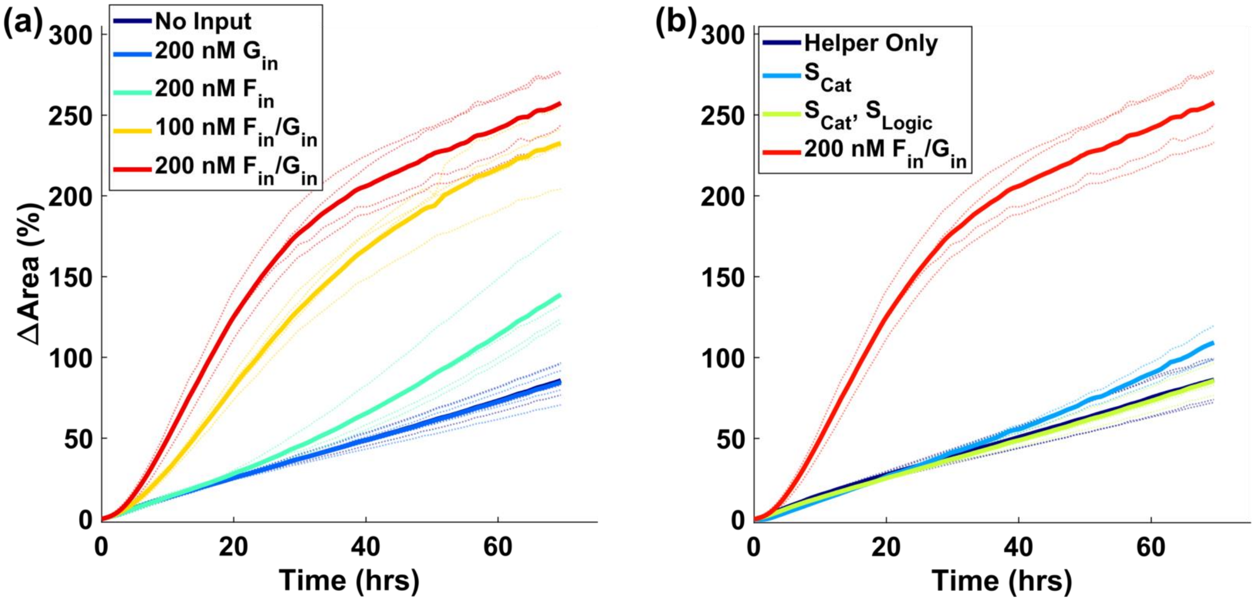

Supplementary Figure 23: Swelling kinetics of the particles when incubated with the Logic Circuit, Helper, and hairpin fuel over 70 hours. (a) Results from the experiment in Figure 5f of the Main Text shown over 70 (rather than 40) hours. (b) Particles swell no more in the presence of Catalyst Source and Logic gate complexes (S_{Cat} and S_{Logic}) at 200 nM and no inputs than they do in the presence of the Helper strand but no circuit components. Solid lines are the averages of measurements of 2 (S_{Cat} in (b)) or 4 particles. Dotted lines are the replicate measurements used to calculate each average.

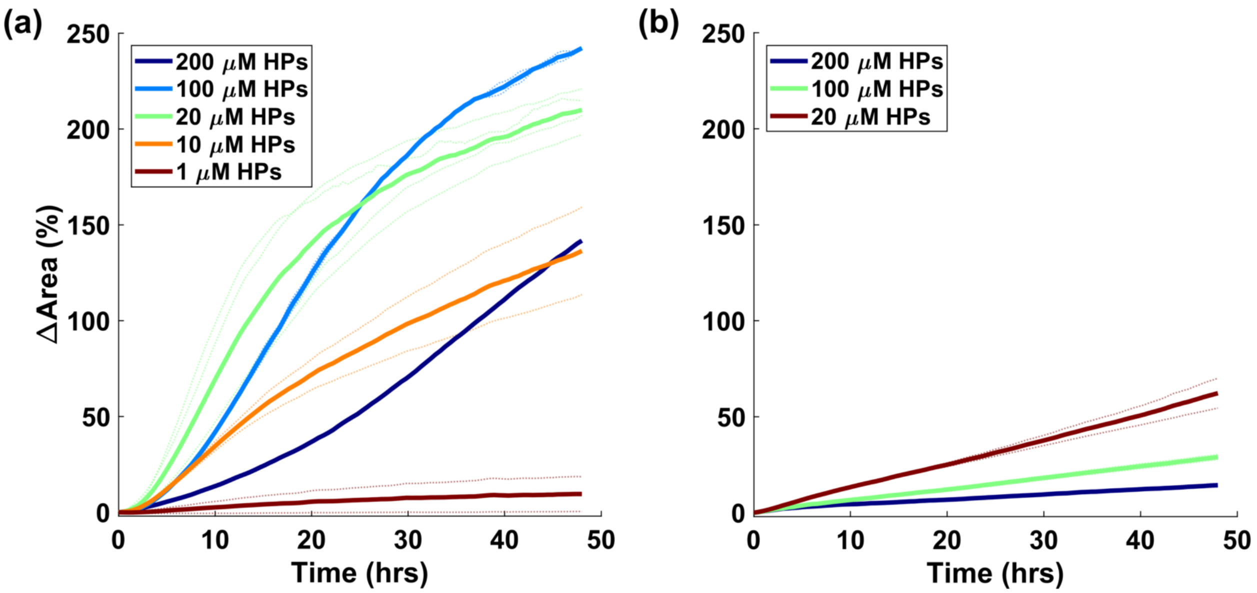

Supplementary Figure 24: Swelling of particles with 100 nM (a) or 10 nM (b) Catalyst and 10 μM Helper strands incubated with different concentrations of hairpin monomers. Concentrations in the legends are "per hairpin type" of which 10% were terminator monomers. Solid lines are single particles (200 μM in (a)) or averages of measurements of 4 (20 μM in (a)) or 2 particles. Dotted lines are the replicate measurements used to calculate each average. When either 10 or 100 nM of Catalyst is present, very high concentrations of hairpin monomers led to slower swelling. Higher hairpin monomer concentrations would be expected to lead to faster swelling, because the rate of their incorporation at unlocked crosslinks should be faster than at lower concentrations. The decreased speed of swelling at higher concentrations of hairpin, could be due to sequestration of Catalyst and/or Helper strands by the hairpins, as their active site domains have complementary regions (Supplementary Figs. 2 and 9).

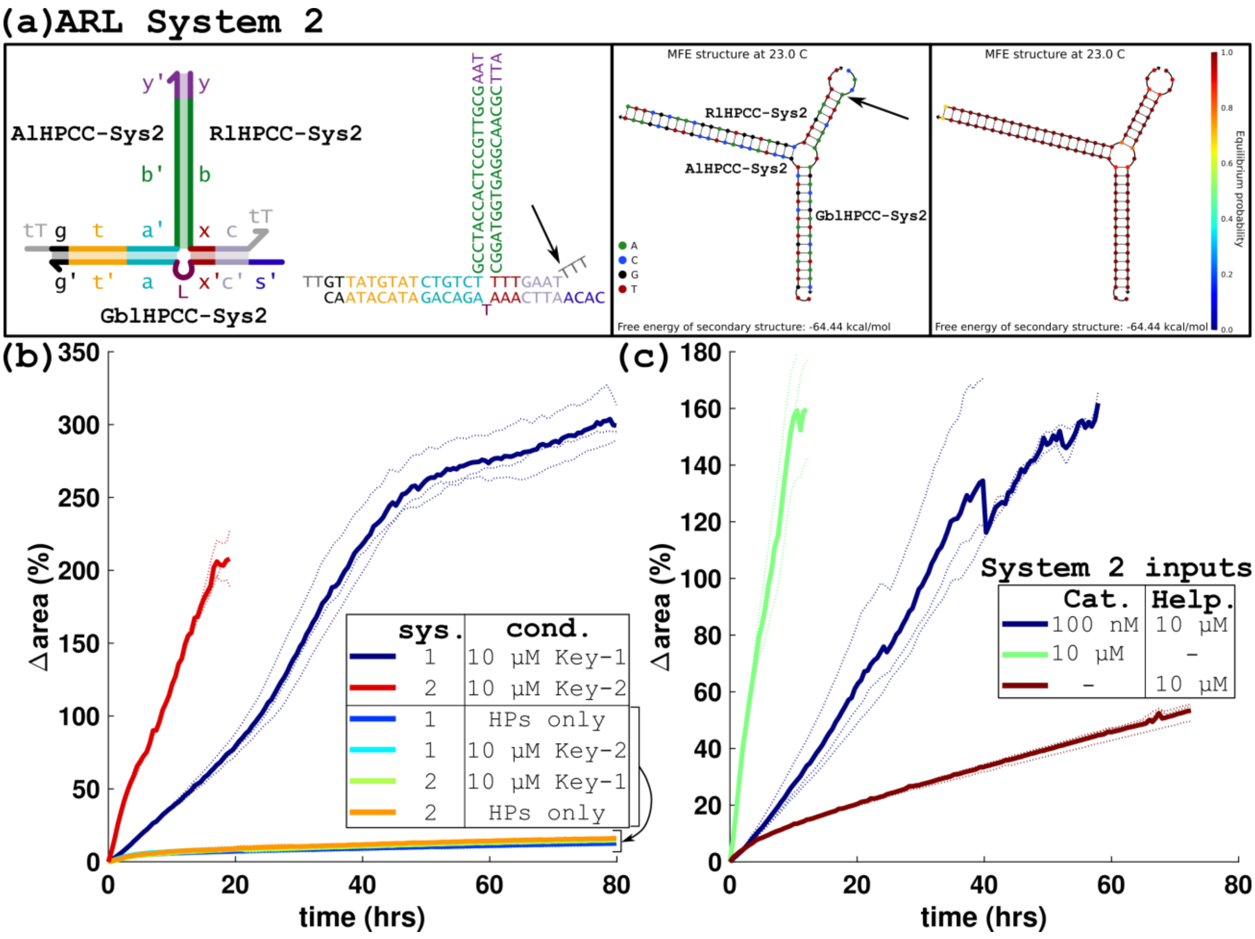

Supplementary Figure 25: Activating hydrogels with locked crosslinks is sequence-specific. (a) Sequences, structure, and equilibrium hybridization probability of a locked crosslink designed to be unlocked by a System 2 unlocking strand and actuated by System 2 hairpin fuel strands (Supplementary Table 1). System 2 and System 1 locked crosslinks should only respond to their respective unlocking and fuel strands, allowing differential control of different hydrogel domains. The lower panes show the secondary structure of the locked crosslink predicted by NUPACK.[1] The arrows point to potential hybridization between the Catalyst-binding toehold of the locking strand and one nucleotide of the 3' termini thymine extension of the R strand. This binding could reduce the effective length of the toehold on the locking strand, potentially reducing the rate of unlocking. The sequences of the System 2 unlocking strands were adapted from previous studies[2] using NUPACK (Supplemental Methods).[1] (b) Hydrogels with locked

System 1 crosslinks swell in response to System 1 Key but not in response to System 2 Key, and hydrogels with locked System 2 crosslinks swell in response to System 2 Key but not in response to System 1 Key. Hairpin fuel, 10% of which were terminating monomers, were added immediately before Key strands and the start of imaging. System 1 hairpins were added to System 1 hydrogels, and System 2 hairpins to System 2 hydrogels. After 20 hours, the edges of hydrogels activated with System 2 Key could not be resolved. Solid lines are averages of measurements of 3 particles. Dotted lines are replicate measurements used to calculate each average. (c) Hydrogels with locked System 2 crosslinks swell in response to System 2 Catalyst/Helper. Hydrogels were incubated with System 2 hairpin fuel, 10% of which were terminating monomers (Supplementary Table 1) for 19 hours prior to adding Catalyst and/or Helper strands. The edges of hydrogels activated with 10 µM System 2 Catalyst could not be resolved after 13 hours and the edges of hydrogels activated with 100 nM Catalyst/10 µM Helper could not be resolved after 40-60 hours. Solid lines are averages of measurements of 4 (10 µM Helper) or 3 particles. Dotted lines are replicate measurements used to calculate each average.

Supplementary References

1. Zadeh, J. N. *et al.* NUPACK: Analysis and design of nucleic acid systems. *J. Comput. Chem.* **32,** 170–173 (2011).
2. Cangialosi, A. *et al.* DNA sequence–directed shape change of photopatterned hydrogels via high-degree swelling. *Science* **357,** 1126–1130 (2017).
3. Seelig, G., Soloveichik, D., Zhang, D. Y. & Winfree, E. Enzyme-free nucleic acid logic circuits. *Science* **314,** 1585–1588 (2006).
4. Zhang, Z., Birkedal, V. & Gothelf, K. V. Enzyme-free colorimetric detection systems based on the DNA strand displacement competition reaction. *New J. Phys.* **18,** 055002 (2016).
5. Qian, L. & Winfree, E. Scaling up digital circuit computation with DNA strand displacement cascades. *Science* **332,** 1196–1201 (2011).
6. Yao, D. *et al.* Integrating DNA-Strand-Displacement Circuitry with Self-Assembly of Spherical Nucleic Acids. *J. Am. Chem. Soc.* **137,** 14107–14113 (2015).
7. Zhang, D. Y., Turberfield, A. J., Yurke, B. & Winfree, E. Engineering entropy-driven reactions and networks catalyzed by DNA. *Science* **318,** 1121–1125 (2007).
8. Zhang, D. Y. & Winfree, E. Control of DNA Strand Displacement Kinetics Using Toehold Exchange. *J. Am. Chem. Soc.* **131,** 17303–17314 (2009).
9. Srinivas, N. *et al.* On the biophysics and kinetics of toehold-mediated DNA strand displacement. *Nucleic Acids Res.* **41,** 10641–10658 (2013).
10. SantaLucia, J. A unified view of polymer, dumbbell, and oligonucleotide DNA nearest-neighbor thermodynamics. *Proc. Natl. Acad. Sci.* **95,** 1460–1465 (1998).
11. Rehman, F. N. *et al.* Immobilization of acrylamide-modified oligonucleotides by co-polymerization. *Nucleic Acids Res.* **27,** 649–655 (1999).
