# Supplementary material for: Modular DNA strand-displacement controllers for directing material expansion
Source: Nat Commun. 2018 Sep 14;9:3766. doi: 10.1038/s41467-018-06218-w (PMC6138645; doi:10.1038/s41467-018-06218-w)
Supplement: Supplementary file 3 — Description of Additional Supplementary Files [file 41467_2018_6218_MOESM3_ESM.docx]

**Description of Additional Supplementary Files**

File Name: Supplementary Movie 1

Description: DNA-crosslinked hydrogel swelling truth table. Significant hydrogel swelling is induced only when both inputs are present. Controller is shown in Figure 5d. Measurements of swelling kinetics are shown in Figure 5f. Logic gate: 200 nM, Catalyst Source: 200 nM, Helper: 10 µM, F_in_ and G_in_, if present: 200 nM.
